# The *Arabidopsis thaliana* core splicing factor PORCUPINE/SmE1 requires intron-mediated expression

**DOI:** 10.1371/journal.pone.0318163

**Published:** 2025-03-26

**Authors:** Varvara Dikaya, Nelson Rojas-Murcia, Ruben Maximilian Benstein, Wolf L. Eiserhardt, Markus Schmid

**Affiliations:** 1 Umeå Plant Science Centre, Department of Plant Physiology, Umeå University, Umeå, Sweden; 2 Department of Plant Biology, Linnean Center for Plant Biology, Swedish University of Agricultural Sciences, Uppsala, Sweden; 3 Department of Biology, Aarhus University, Aarhus, Denmark; 4 Royal Botanic Gardens, Kew, Richmond, Surrey, United Kingdom; Abdul Wali Khan University Mardan, PAKISTAN

## Abstract

Plants are prone to genome duplications and tend to preserve multiple gene copies. This is also the case for the genes encoding the Sm proteins of *Arabidopsis thaliana* (L). The Sm proteins are best known for their roles in RNA processing such as pre-mRNA splicing and nonsense-mediated mRNA decay. In this study, we have taken a closer look at the phylogeny and differential regulation of the SmE-coding genes found in *A. thaliana*, *PCP/SmE1*, best known for its cold-sensitive phenotype, and its paralog, *PCPL/SmE2*. The phylogeny of the *PCP* homologs in the green lineage shows that *SmE* duplications happened multiple times independently in different plant clades and that the duplication that gave rise to *PCP* and *PCPL* occurred only in the Brassicaceae family. Our analysis revealed that *A. thaliana* PCP and PCPL proteins, which only differ in two amino acids, exhibit a very high level of functional conservation and can perform the same function in the cell. However, our results indicate that *PCP* is the prevailing copy of the two *SmE* genes in *A. thaliana* as it is more highly expressed and that the main difference between *PCP* and *PCPL* resides in their transcriptional regulation, which is strongly linked to intronic sequences. Our results provide insight into the complex mechanisms that underlie the differentiation of the paralogous gene expression as an adaptation to stress.

## Introduction

Rooted in place, plants cannot escape changes in their environment. Instead, they constantly monitor their surroundings and adjust their growth and physiology to ensure survival. Daily and seasonal temperature fluctuations in particular are important environmental cues that, together with other external signals, inform plants about their surroundings. The actual responses differ depending on the temperature the plant experiences. For example, moderately elevated temperature triggers a reaction known as thermomorphogenesis whereas severe heat stress can stop plant growth altogether and ultimately result in the death of the plant [1,2]. Similarly, cold is usually divided into low permissive temperatures that only mildly affect plant development, chilling that induces a cold and general stress response, and temperatures below 0°C, or freezing, that might cause ice formation and severe damage inside the plant cell. Importantly, exposure to chilling conditions can prime and prepare plants for a further temperature drop [3,4]. Depending on their habitat, plants may face either only chilling, freezing, or both.

Perception of temperature in plants is mediated by a combination of multiple signaling pathways rather than a single dedicated sensor. Initially, temperature modulates the physical properties of cellular components such as the rigidity of membranes, enzymatic activity, conformational changes of protein and nucleic acid structures, (dis-) assembly of the cytoskeleton, and changes in the viscosity of solutions [5–8]. These primary physical responses initiate signals via multiple pathways that work simultaneously: MAPK cascades, Ca^2 +^ influx into the cell, and rearrangement of transcription and translation [9]. Many players of cold signaling, e.g., phytochromes, abscisic acid, and reactive oxygen species, are not specific to temperature responses but also participate in other signal pathways such as light, osmotic stress, drought, and general stress response [10–12]. The ultimate result of the signal transmission is an adjustment of the gene regulation, metabolism, overall developmental program of the plant, and its acclimatization to the current growth conditions. Not surprisingly given the scope and importance of temperature regulation, multiple mechanisms, including regulation at the epigenetic level, transcription, translation, and post-translational modifications, contribute to this process [11].

RNA splicing [13,14], the process during which non-coding introns are removed from the pre-mRNA and exons are joined, has emerged as a key regulatory mechanism that enables plants to respond to and adjust their physiology, growth, and development to their surroundings [15–18]. Conceptually, RNA splicing is often divided into constitutive splicing, during which a prevalent RNA isoform is formed, and alternative splicing (AS), which enables the organism to produce multiple distinct mature mRNAs from one pre-mRNA [19,20]. In *Arabidopsis thaliana*, about 60% of genes undergo AS [21–23].

AS has been shown to play a role in establishing organ identity, regulating transitions between developmental stages, and contributing to the response of plants to various environmental stimuli [24–26]. For example, it has been shown that the number and type of AS events in the cell change significantly in response to cold and approximately 27% of the cold-responsive genes showed differential AS in *A. thaliana* [27]. However, even though multiple observations demonstrate the importance of RNA splicing in coordinating a plant’s response and acclimation to temperature [28–30], the contribution of individual splicing factors and their connection to canonical temperature signaling pathways is largely unknown [31].

A gene that has recently been shown to be important for the proper growth and development of *A. thaliana*, particularly under cool ambient temperatures and in cold conditions, is *PORCUPINE* (*PCP*; *SmE1*; At2g18740) [32–34]. *PCP* encodes an E-subunit of the evolutionary conserved Sm-ring that together with U-rich small nuclear RNAs forms the core of the U1, U2, U4, and U5 small nuclear ribonucleoproteins (snRNPs) [35,36]. All members of the Sm-ring are highly conserved in their structure and possess an N-terminal α-helix followed by a five-stranded antiparallel β-sheet which forms characteristic Sm1 and Sm2 motifs [35,37,38]. Structural analyses of the human Sm-ring revealed that the ring-like structure is formed by the interaction between the β4 strand of one Sm-protein and the β5 strand of its counterclockwise neighbor [39]. The Sm-site that binds snRNA inside the central opening of the Sm-ring is formed by residues that are positioned in the loops L3 (between strands β2 and β3) and L5 (between β4 and β5) and belong to the Sm1 and Sm2 motif, respectively [39]. Together with other proteins, snRNPs form the core of the spliceosome, the molecular machinery that catalyzes the splicing reaction [36]. An intriguing feature of *A. thaliana* plants deficient in *PCP* is that these mutants appear macroscopically like wild-type plants at 23°C but display severe developmental phenotypes at moderately cool temperatures of 16°C including significant aberrations in shoot- and root apical meristem development, delayed flowering time, and male sterility [32]. More recently, it has been shown, however, that the *pcp-1* mutant exhibits subtle phenotypes at the cellular level, for example, in the root meristem, when grown at a temperature of 23 °C [40]. These findings demonstrate that the *PCP* gene is important for normal development across the entire temperature range, but that mutant phenotypes primarily manifest at cooler temperatures.

*Sm* genes have been duplicated in plants, and often two or more copies of each subunit are found in a given species [41,42]. This is also the case in *A. thaliana* where each of the seven subunits that form the Sm-ring has been duplicated [43]. The coding sequences of *A. thaliana PCP* (SmE1) and its paralog *PCP-LIKE* (PCPL; SmE2) are highly similar and the two proteins differ in only two amino acids [32]. The biological function of PCPL has, however, not yet been investigated in detail.

Here we have conducted a detailed phylogenetic analysis of SmE proteins in green plants (Viridiplantae). It revealed that duplication of SmE likely occurred multiple times and independently in different clades of the Viridiplantae. A comparison of the predicted structure of the two *A. thaliana* SmE proteins suggested that two amino acid differences that distinguish PCP and PCPL are unlikely to affect their function. In contrast to mutations in *PCP*, *pcpl* mutant plants did not display any phenotypes under any of the temperatures tested, even though *PCPL* gene expression is modulated by temperature. Functional analyses indicate that the two *A. thaliana* SmE proteins can, in principle, fulfill the same function, and differences reside mostly in their transcriptional regulation. Importantly, the expression of *PCP* was found to be strongly linked to its introns. Among them, the 1^st^ intron might be of particular importance and its deletion can have a strong effect on *PCP* expression. However, other introns also seem to contribute to the regulation of *PCP* expression. These findings suggest that *cis*-regulatory elements distributed across multiple introns or complex interactions between non-coding and coding sequences during transcription and RNA processing ensure timely and correct expression of *PCP* in response to temperature.

## Results

### *SmE* duplication is a reoccurring event throughout plant evolution

The *Arabidopsis thaliana* genome encodes two SmE proteins, AtPCP (SmE1; At2g18740) and AtPCPL (AtPCP-like; SmE2; At4g30330). To reconstruct the evolution of the *SmE* paralogs we retrieved *SmE* gene and protein sequences from 24 species of green plants. These represented major lineages such as green algae (*Chlamydomonas reinhardtii*), bryophytes (*Physcomitrium patens*), lycophytes (*Selaginella moellendorffii*), and ferns (*Ceratopteris richardii*). Most of the *SmE* genes and proteins, however, belong to the angiosperms, including *Amborella trichopoda* (the sister species to the rest of angiosperms), as well as several monocotyledonous species and representatives of two major Eudicot clades, namely the Asterids and Rosids. Five Brassicaceae species were added to the analysis to increase local resolution in proximity to *A. thaliana*. Out of these 24 species, two, *Chlamydomonas reinhardtii* and *Ceratopteris richardii*, possessed only one *SmE* gene while in the remaining species between 2 to 6 paralogs of *SmE* could be identified (S1 Table).

**Fig 1:**
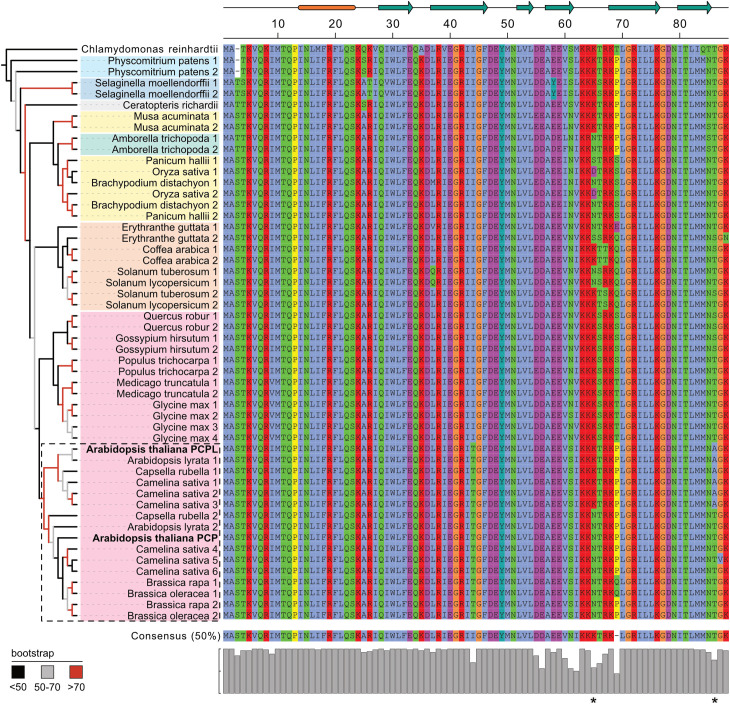
Phylogenetic tree of *SmE* genes and corresponding SmE protein alignment. A maximum likelihood tree based on the CDS alignment was constructed using IQ-TREE software. Nodes are colored according to the bigger clades they belong to: green algae (no color), Bryophytes (light blue), Lycophytes (dark blue), Ferns (grey), Monocots (yellow), Eudicots (orange for Asterids and magenta for Rosids). The Brassicaceae family is highlighted in a dashed box. Protein sequences are colored according to the ClustalX color scheme [45]. *SmE* copies are numbered for each species except *Arabidopsis thaliana*, where gene names *PCP* and *PCPL* are given instead and highlighted in bold. Corresponding gene IDs can be found in S1 Table. The schematic drawing above the protein alignment represents the secondary structure of the protein. The N-terminal alpha-helix is indicated in orange, and teal arrows mark beta-strands. Asterisks mark amino acid positions 65 and 86 that discriminate PCP (SmE1) and PCPL (SmE2).

Initial alignment of the genomic DNA sequences showed very high conservation within the exon sequences only. The low sequence similarity of the introns as well as a big variability of their lengths prevented the construction of a reliable phylogenetic tree based on the genomic DNA alignment. Instead, we proceeded with reconstructing a SmE phylogeny using amino acid and cDNA sequence alignments. The phylogenetic tree overall mapped to the currently accepted evolutionary tree of angiosperms [44] (Fig 1). *SmE* genes did not split into two distinct groups, *SmE1-like* (including *AtPCP*) and *SmE2-like* (including *AtPCP-like*), rejecting our initial hypothesis that *A. thaliana PCP* and *PCPL* were the results of an ancient gene duplication in the green lineage. Instead, our results suggest that *SmE* duplications occurred multiple times independently in various branches of the green lineage and that the *SmE* duplication that led to *AtPCP* and *AtPCPL* appears to be specific to the Brassicaceae family. Interestingly, we did not observe *SmE2-like* genes in *Brassica rapa* and *Brassica oleracea*, which instead contained additional copies of *SmE1*, suggesting the secondary loss of *PCPL-like* genes and additional *PCP* duplication in the *Brassica* genus. Similar duplication events that were inherited by a whole clade of closely related species occurred in grasses (Poaceae) and presumably in the Solanaceae family. Duplications observed in the remaining examined species did not group inside clades above the species level.

### Brassicaceae-specific amino acid substitutions are unlikely to affect SmE protein function

Our phylogenetic analysis indicated that *A. thaliana PCP* and *PCPL* are the result of a Brassicaceae-specific duplication. Specifically, the SmE1-like and SmE2-like proteins differ in two amino acids, an asparagine to lysine substitution in position 65 and a threonine to alanine substitution in position 86. The question arises if these amino acid substitutions affect the function of the proteins.

Both amino acids map to regions that are unlikely to play crucial roles in protein folding and affect its function. Position 65 falls into the loop region between two beta-strands or the very beginning of the beta-strand, and position 86 lies in the unstructured C-terminal region of the protein after the final predicted beta-strand. Furthermore, substitutions in position 65 are quite diverse in their physical properties and occurred independently several times in the green lineage (Fig 1). In contrast, substitutions in position 86 are not as widespread, but the substitution of threonine (T) in PCP by alanine (A) in PCPL could in principle affect post-translational protein modifications, in particular phosphorylation. Nevertheless, considering the position and nature of the two amino acid substitutions they seem unlikely to affect protein function. This notion is supported by the predicted structures of PCP and PCPL, which are largely congruent (S1 Fig). In particular, the substitutions in positions 65 and 86 do not appear to affect the protein backbone and structure (S1 Fig).

### SmE proteins are essential for plant development

Loss of PCP (SmE1) in *A. thaliana* has been reported to result in temperature-sensitive phenotypes. *pcp-1* was initially described as a mutant sensitive to cold (4°C) and cool ambient temperature of 16°C [32,33]. In agreement with these previous reports, we observed an intermediate phenotype with drastic shortening of the root and anthocyanin accumulation in the cotyledons in *pcp-1* grown at 10°C (S3 Fig). We also observed a mild delay in the development of 10-day-old *pcp-1* at 23°C. Mutants had smaller leaf rosettes and shorter roots with less abundant and developed lateral roots in comparison to Col-0 (S4 Fig). This delay in development appeared to be transient, and *pcp-1* plants eventually caught up and developed similarly to WT at 23°C [32]. Confirming its cold temperature-sensitive nature, 7-day-old *pcp-1* seedlings grown at 27°C did not exhibit significant alterations in shoot and root development compared to Col-0 plants growing in the same conditions (S3 Fig).

**Fig 2:**
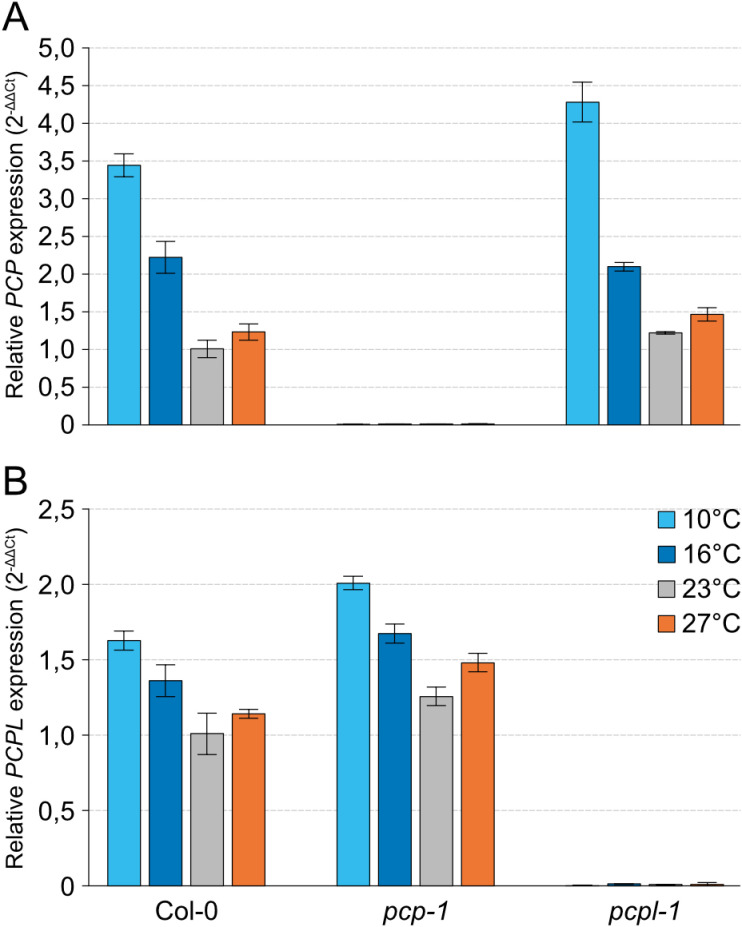
Expression of *PCP* and *PCPL* in *A. thaliana SmE* mutants. *PCP* (*SmE1*) (A) and *PCPL* (*SmE2*) (B) expression was determined in the Col-0, *pcp-1*, and *pcpl-1* grown at 10°C, 16°C, 23°C and 27°C by RT-qPCR. Bars show the mean expression based on 3 biological replicates with 3 technical replicates each. Error bars indicate the standard deviation (SD).

Consistent with the cold-sensitivity of the *pcp-1* mutants, *PCP* expression in the WT was induced two-fold at 16°C and almost 3.5-fold at 10°C in comparison to 23°C (Fig 2A). We also observed a mild induction of *PCP* in response to elevated temperature. Interestingly, our qPCR data showed that *PCPL* in WT displayed the same overall expression profile as *PCP* (Fig 2B) but the expression changes in response to cool and warm temperatures were much weaker. For example, whereas *PCP* expression was induced more than 3-fold at 10°C when compared to 23°C, *PCPL* expression increased only 1.5-fold. These findings suggested that *PCP* might be the more important of the two *SmE* genes in response to temperature.

To test this hypothesis, we generated two *pcpl* mutant alleles using CRISPR/Cas9 mutagenesis. Interestingly, we found that *pcpl-1* and *pcpl-2* mutants were indistinguishable from wild-type across a wide range of temperatures (S3 and S4 Figs), which is remarkably different from the temperature-sensitive *pcp-1* (S3 and S4 Figs). Possibly, the expression of *PCPL* is sufficient to compensate for the loss of *PCP* in *pcp-1* at 23°C and higher temperatures, but *PCPL* is not enough to fulfill the splicing needs of the cell at lower temperatures. Alternatively, there might be differences in the processing of the transcripts or even in the proteins themselves.

**Fig 3:**
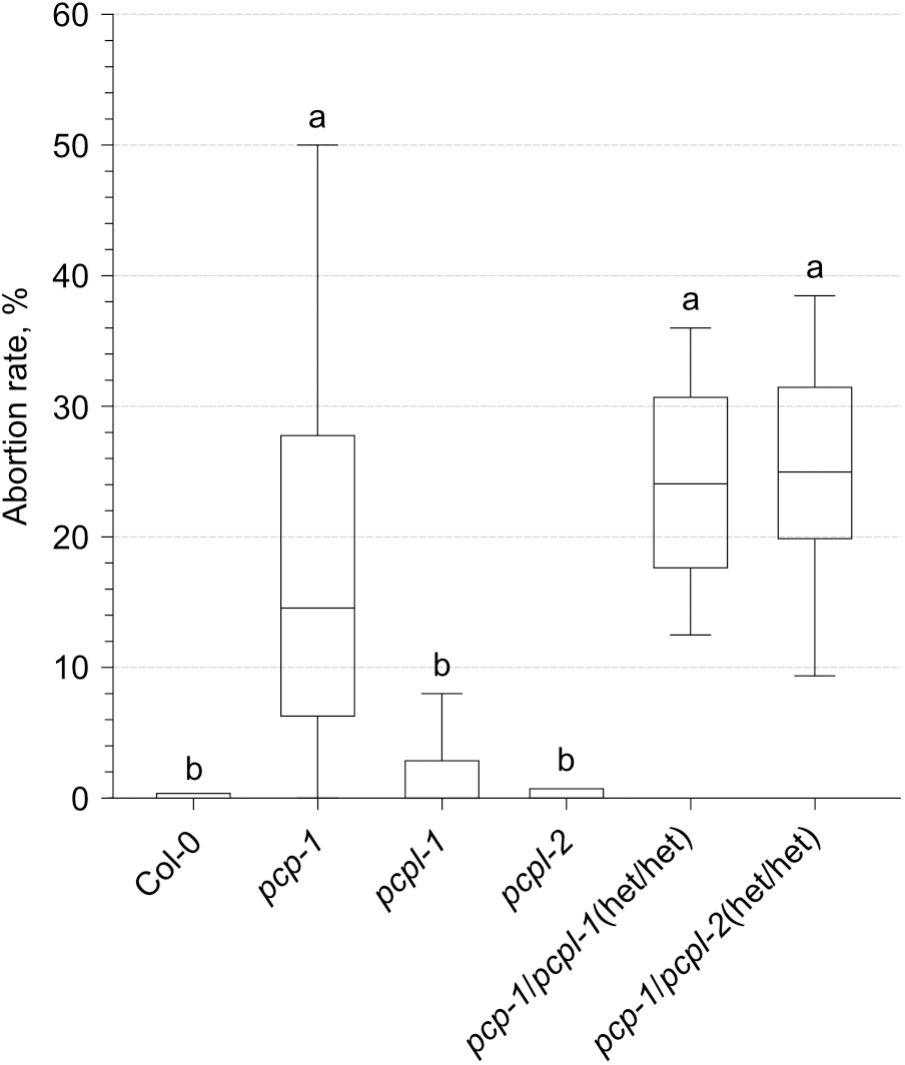
Seed abortion rate of *SmE* mutants. The fraction of aborted ovules relative to the total number of ovules in the siliques (n =  20) of the six different genotypes was calculated and plotted as a box-with-whiskers plot. The box spans over the 25^th^ to 75^th^ percentiles, the line inside the box represents the median, whiskers reach out to the minimal and maximal values in each dataset. A one-way ANOVA test with Tukey correction was performed after the removal of outliers using the ROUT method (Q =  1%). All statistical tests were run using GraphPad Prism. Letters represent significantly different (P <  0.05) groups.

However, plants clearly require at least one functional copy of *SmE* as demonstrated by the finding that 25% of the developing seeds of self-pollinated double-heterozygous *pcp pcpl* plants were aborted (Fig 3), indicating gametophytic lethality. The same experiment also revealed a moderately increased seed abortion rate in the *pcp-1* single mutant. In contrast, the seed set was essentially unaffected in the *pcpl* single mutants (Fig 3), again highlighting phenotypic differences between the mutants in these paralogous genes.

### *PCP* expression requires genomic and intronic sequences

Since phylogenetic and structure-function analyses had not revealed any major differences in PCP and PCPL proteins, the question arises as to why the two mutants have such different phenotypes. Hypothesizing that the reason might reside in the transcriptional regulation of the genes, constructs expressing either the CDS (from start codon to stop codon), to which we refer as *cPCP* and *cPCPL*, respectively, or the genomic DNA (i.e., exons plus introns) of *PCP* (*gPCP*) and *PCPL* (*gPCPL*) under the regulation of the *PCP* promoter (*pPCP*) and terminator (*tPCP*) sequences were created. In addition, constructs overexpressing the CDS of either of these two genes under the control of the constitutive *35S* promoter (S2 Table) were also generated.

**Fig 4:**
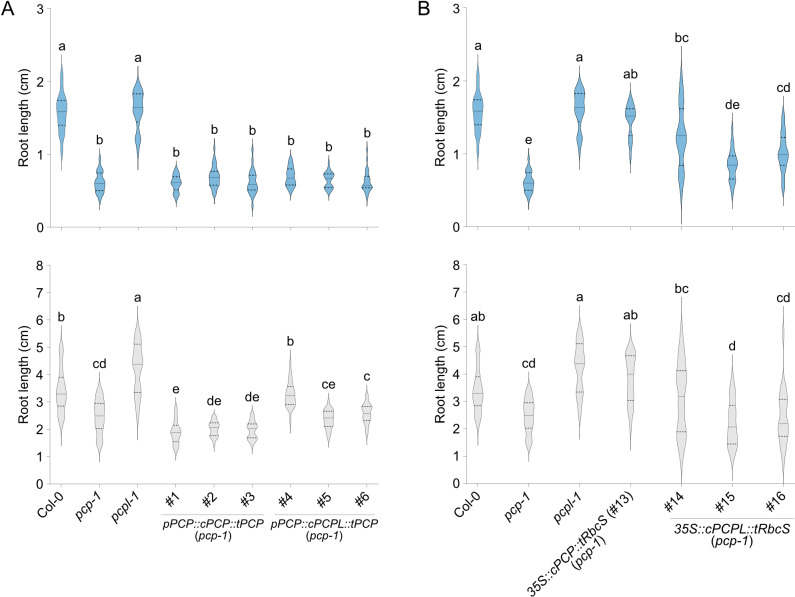
Root length of lines expressing *SmE* coding sequences. (A) Root phenotype of three independent lines carrying *pPCP::cPCP::tPCP* (#1-3) and *pPCP::cPCPL::tPCP* (#4-6) constructs in the *pcp-1* background. Col-0, *pcp-1*, and *pcpl-1* serve as controls. (B) Root phenotype of three independent lines carrying *35S::cPCPL::tRbcS* (#14-16) constructs in the *pcp-1* background. Col-0, *pcp-1*, *pcpl-1*, and previously established *35S::cPCP::tRbcS* (#13) [32]. The length of the primary root of 10-day-old seedlings grown at 16°C (blue) and 23°C (light grey) is shown as violin plots (n =  20 to 25). Solid lines represent the median and dashed lines represent quartiles. One-way ANOVA test with Tukey correction was performed using GraphPad Prism. Letters represent significantly different (P <  0.05) groups.

**Fig 5:**
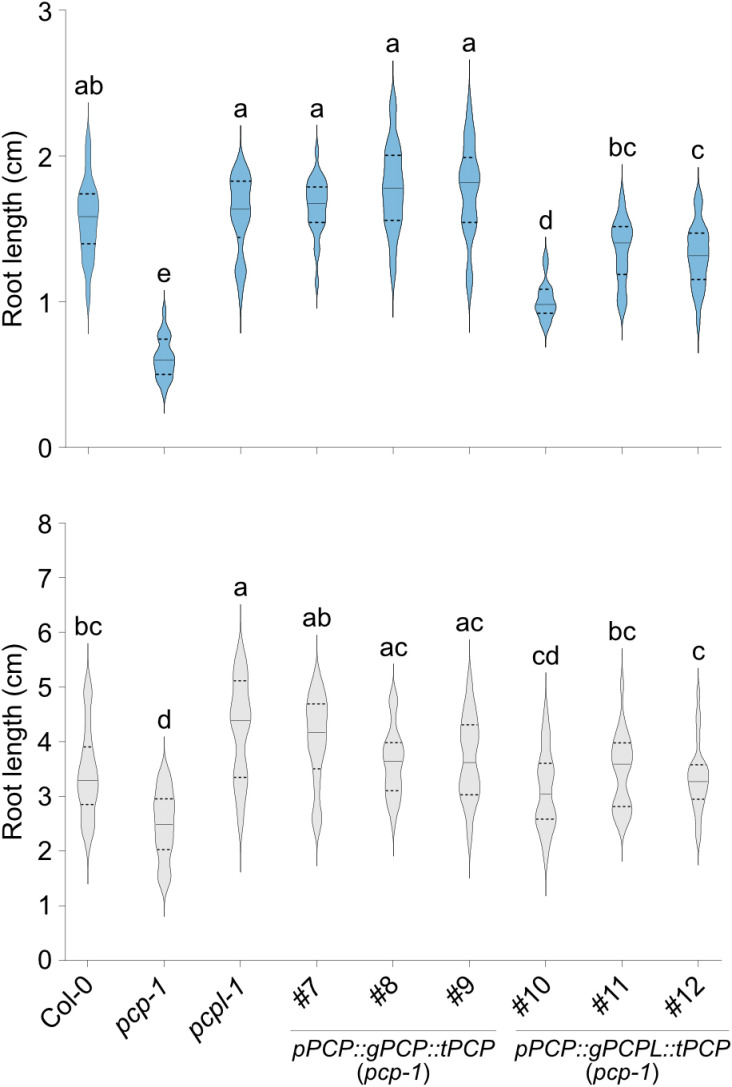
Genomic *PCP* construct rescues the root length phenotype of *pcp-1.* Length of the primary root of three independent lines in the *pcp-1* background transformed with *pPCP::gPCP::tPCP* (#7-9) and *pPCP::gPCPL::tPCP* (#10-12) constructs. Col-0, *pcp-1*, and *pcpl-1* serve as controls. Root length was determined using 10-day-old seedlings grown at 16°C (blue) and 23°C (light grey). Root lengths are shown as violin plots (n =  20 to 25) where a solid line represents the median and dashed lines represent the quartiles. A one-way ANOVA test with Tukey correction was performed using GraphPad Prism. Letters represent significantly different (P <  0.05) groups.

**Fig 6:**
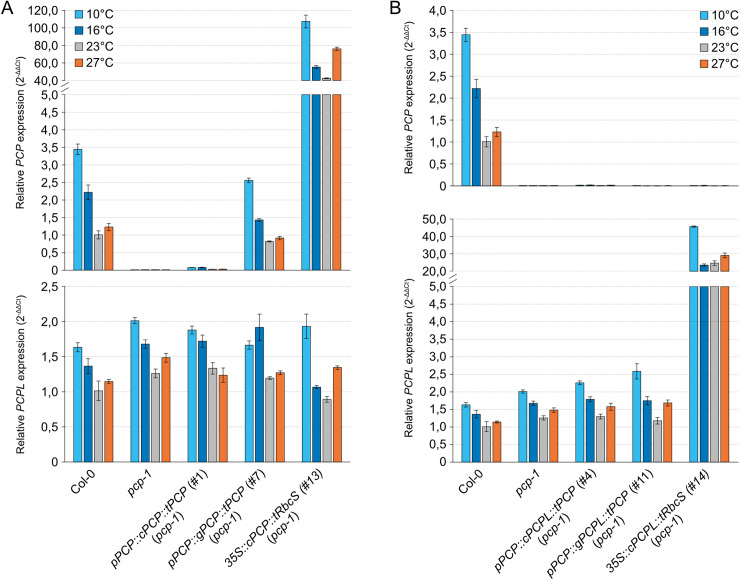
Expression analysis of *PCP* and *PCPL.* (A) Expression of *PCP* and *PCPL* in Col-0, *pcp-1*, and lines carrying *pPCP::cPCP::tPCP* (#1), *pPCP::gPCP::tPCP* (#7), and *35S::cPCP::tRbcS* (#13) [32] constructs in the *pcp-1* background. (B) Expression of the *PCP* and *PCPL* in Col-0, *pcp-1*, and *pcp-1* lines carrying *pPCP::cPCPL::tPCP* (#4), *pPCP::gPCPL::tPCP* (#11), and *35S::cPCPL::tRbcS* (#14). Gene expression was determined in seedlings after 5 days of growth at 23°C followed by 24 h of growth at 10°C (light blue), 16°C (dark blue), 23°C (grey), and 27°C (orange). Bars show the mean expression calculated from three biological replicates with three technical replicates each. Error bars indicate the standard deviation (SD).

These constructs were introduced into the *pcp-1* mutant and primary root length and overall morphology were used as a measure of phenotypic rescue. Interestingly, at temperatures between 10°C to 23°C lines (#1-3) carrying the *pPCP::cPCP::tPCP* construct exhibited a *pcp-1*-like phenotype, which got more severe at lower temperatures (Fig 4A, S3 and S4 Figs). At the same time, *p35S*-mediated overexpression of the *PCP* CDS resulted in full phenotype restoration (Fig 4B, S3, and S4 Figs). Similarly, *pPCP::gPCP::tPCP* constructs (#7-9) fully rescued the *pcp-1* phenotype (Fig 5, S3, and S4 Figs). Consistent with the phenotyping data, RT-qPCR showed that in the case of the gDNA and overexpression lines *PCP* expression was restored to approximately 70% of the WT levels and strongly overexpressed, respectively (Fig 6A). Importantly, the expression of *pPCP::gPCP::tPCP* also restored the temperature-dependent expression of *PCP*, which might indicate post-transcriptional regulation in addition to transcriptional regulation via *cis*-regulatory elements present in the genomic sequence of *PCP*. In contrast, no expression of *PCP* beyond the residual expression typical for the *pcp-1* mutant could be detected in the lines expressing the *PCP* CDS under the control of *pPCP* and *tPCP*. Taken together, these findings support the idea that phenotype restoration correlates with the expression of *PCP*, and that the full gDNA sequence is essential for proper expression.

### Transcription regulation differs between *PCPL* and *PCP*

Similarly, expression of the *PCPL* CDS under the control of the *pPCP* and *tPCP* (lines #4-6) was unable to rescue the *pcp-1* phenotype at 16°C and 10°C (Fig 4A, S3, and S4 Figs). Except for line #4, which showed a moderate phenotype rescue, all lines were statistically indistinguishable from *pcp-1* also at 23°C, while RT-qPCR analysis detected mild elevation in the *PCPL* expression at all temperature conditions (Fig 6B).

Lines #10-12, which expressed the *gPCPL* construct including all introns under the control of the *PCP* promoter and terminator (*pPCP::gPCPL::tPCP*) showed partial to full phenotypic rescue of the *pcp-1* phenotype at 16°C and 23°C (Fig 5 and S3 Fig). However, irrespective of temperature (10°C to 23°C) these lines performed worse than their counterparts (lines #7-9) expressing *gPCP* under the control of the *PCP* promoter and terminator (Fig 5, S3, and S4 Figs). Expression of *PCPL* in line #11 was upregulated in comparison to Col-0 at all temperatures, especially at 10°C. *PCPL* expression did not differ significantly between #11 and *pcp-1* except at 10°C (Fig 6B). Together, these findings suggest a discrepancy between the regulation of *PCP* and *PCPL* expression, likely due to different regulatory elements in the gDNA sequences of *PCP* and *PCPL*.

Overexpression of *cPCPL* (lines #14-16) partially rescued the *pcp-1* phenotype, resulting in an overall root morphology that more closely resembled WT than *pcp-1* (S4 Fig). However, the rescue of root length in these *cPCPL* overexpression lines was more variable than that observed in the WT plants and other rescue lines, often falling between WT and *pcp-1* (Fig 4B). Possibly, this variability is a result of the very high overexpression of *PCPL* in these lines, which vastly exceeds the endogenous *PCPL* levels. Such misregulation of a central splicing factor can be expected to have unwanted effects on downstream genes, which could result in the observed high phenotypic variability. Interestingly, we also observed that the expression of *PCP* and *PCPL* in the overexpression lines was mildly temperature-dependent (Fig 6), confirming previous results that the 35S promoter is modulated by temperature [46].

### PCP and PCPL proteins are functionally redundant

**Fig 7:**
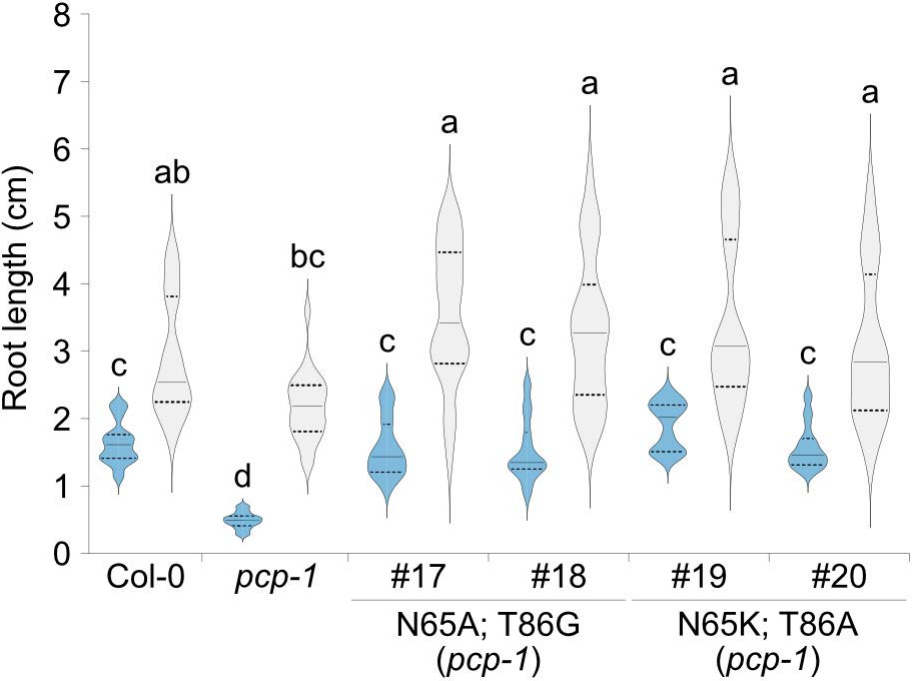
Root length of PCP-PCPL amino acid substitution lines. Length of the primary root of plants carrying *pPCP::gPCP_N65A_T86G::tPCP* (#17-18) and *pPCP::gPCP_N65K_T86A::tPCP* (#19-20) rescue constructs in the *pcp-1* background. Col-0 and *pcp-*1 served as controls. Root length was measured using 10-day-old seedlings grown at 16°C (blue) and 23°C (light grey). Root lengths are shown as violin plots (n =  20 to 25) where the solid line indicates the median and the dashed lines represent the quartiles. A two-way ANOVA test with Tukey correction was performed using GraphPad Prism. Letters represent significantly different (P <  0.05) groups.

Having established a genomic *PCP* construct that not only rescued the phenotype of *pcp-1* at low temperatures but also the temperature-responsive expression enabled us to address the role of the amino acid substitutions that discriminate PCP and PCPL proteins. To this end, the amino acid substitutions found in PCPL (N65K; T86A) were introduced into the *gPCP* genomic rescue construct (*pPCP::gPCP_N65K_T86A::tPCP*; lines #19-20) and introduced into *pcp-1*. We found that this construct fully restored the WT phenotype at 16°C (Fig 7) and 23°C, confirming our previous assumptions that the SmE1 and SmE2 proteins in *A. thaliana* were functionally redundant. Similar results were obtained using a construct in which the two amino acids that distinguish the two SmE proteins were changed to alanine and glycine (*pPCP::gPCP_N65A_T86G::tPCP; lines* #17-18).

### Introns work together to regulate *PCP* expression

**Fig 8:**
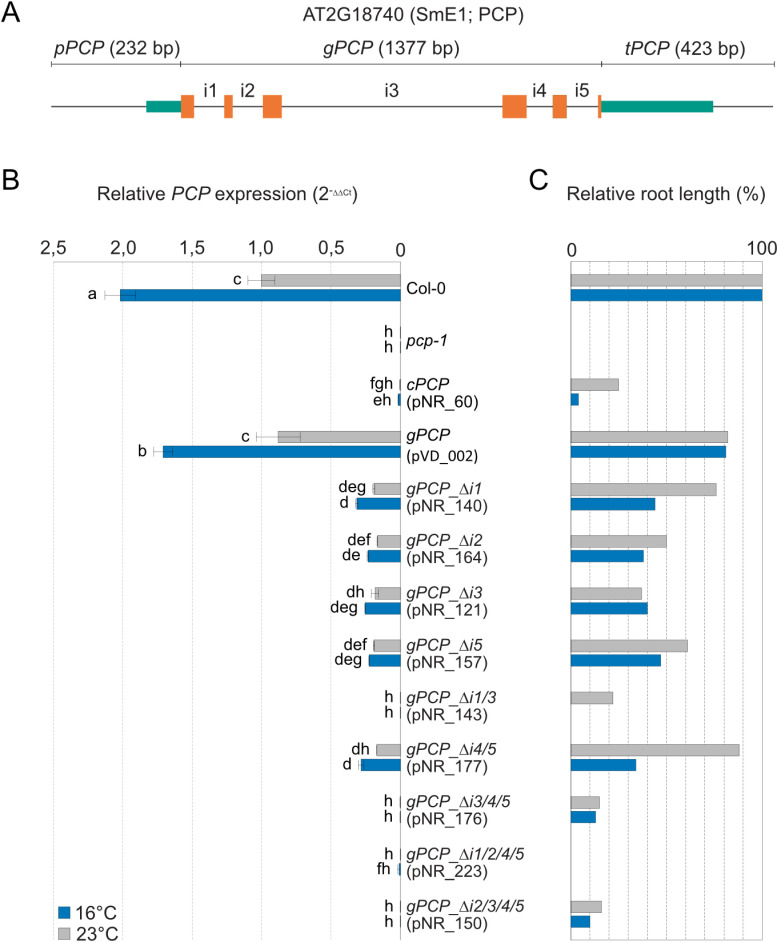
Introns are required for *PCP* expression. (A) Schematic representation of the full genomic *PCP* construct used to perform the intron deletion studies. Teal boxes represent promoter and terminator regions, orange boxes represent exons, and solid lines represent non-coding sequences. i1 to i5 labels indicate introns 1 to 5. (B) Expression of *PCP* in the *pcp-1* mutant transformed with various intron deletion constructs. Deletion lines and constructs are labeled as *gPCP_ ∆ iX*, where *gPCP* represents a full *pPCP::gPCP::tPCP* construct, ∆  stands for deletion, i stands for intron, and X indicates the number(s) of the deleted intron(s). Col-0, *pcp-1*, and a *pcp-1* rescue line carrying the full-length *pPCP::gPCP::tPCP* construct served as controls. Gene expression was determined in seedlings after 5 days of growth at 23°C followed by 24 h of growth at 16°C (blue) or 23°C (grey). Bars show the mean expression calculated from one to three biological replicates with three technical replicates each. Error bars indicate the standard deviation (SD). A two-way ANOVA test with Tukey correction was performed using GraphPad Prism. Letters represent significantly different (P <  0.05) groups. (C) Average root-phenotype rescue relative to Col-0 (100%) and *pcp-1* (0%) in the corresponding lines from panel (B).

Since the full genomic sequence of *PCP* with 5’ promoter and 3’ UTRs was necessary for complete *pcp-1* phenotype rescue, and expression of the *PCP* cDNA under the control of the *PCP* promoter and terminator did not result in expression of the gene, we assumed that *PCP* expression was linked to its introns. To test this hypothesis, several intron-deletion constructs were created in the context of *gPCP* (S2 and S3 Tables), introduced into *pcp-1*, and used to assess *PCP* expression and the extent of phenotypic rescue (Fig 8, S5, and S6 Figs).

Deletion of single introns 2, 3, and 5 (*gPCP- ∆ i2*, *gPCP- ∆ i3*, and *gPCP- ∆ i5*) resulted in a considerable but not complete loss of *PCP* expression (Fig 8B) and a corresponding reduction in the rescue of the primary root length phenotype (Fig 8C and S5 Fig). The combined deletion of introns 4 and 5 (*gPCP-Δi4/5*) largely resembled the deletion of intron 5, suggesting that intron 4 plays only a minor role in regulating *PCP* expression. Interestingly, *PCP* expression in these intron deletion lines still responded to temperature and was induced by 16°C, albeit at a reduced level, suggesting that these introns were not required for the cold-induced expression of *PCP*. For these intron deletion lines, *PCP* expression and phenotypic rescue correlated well and was consistent among independent rescue lines. In contrast, the deletion of intron 1 (*gPCP- ∆ i1*) gave more variable results (Fig 8 and S6 Fig). Of the two individual lines tested, one showed a low expression of *PCP* and phenotype rescue of the root phenotype (Fig 8). The second line showed an even weaker rescue and *PCP* expression was essentially undetectable (S6 Fig). However, when combined with the deletion of intron 3, the deletion of intron 1 (*gPCP-Δi1/3*) invariably leads to the complete loss of *PCP* expression, suggesting that intron 1 plays an important role in regulating *PCP* expression. On the other hand, the presence of intron 1 in the CDS alone (*gPCP-Δi2/3/4/5*) was insufficient to drive the expression and restore the *pcp-1* phenotype (Fig 8 and S5B Fig). Finally, the deletion of more than two introns appears to have an additive effect, resulting in a complete loss of *PCP* expression and a *pcp-1*-like root phenotype at 16°C in all combinations tested (Fig 8 and S5 Fig). Taken together, our results show that introns are essential for the proper *PCP* expression, and they work in tandem. Whether introns 1 takes the role of the main expression switch or promotes *PCP* transcript accumulation by other means remains to be established.

## Discussion

Throughout the course of evolution, plants underwent multiple genome duplications and preserved copies of paralogous genes. Through neo- and sub-functionalization these paralogues either acquired new functions or performed their original tasks under different conditions, thereby increasing the adaptability of plants to adverse environmental conditions [47].

Here we investigated *SmE* genes and proteins to shed light on the evolution of this important regulator of RNA splicing in the green lineage. Consistent with the work by Cao et al. 2011 [43] who had analyzed the relationship among Sm proteins in *A. thaliana*, we found that the duplication resulting in *PCP/PCPL* happened independently of *SmE* duplications in other plant clades during the most recent Brassicaceae-specific whole-genome duplication (WGD) event, namely At-α [48]. In general, reoccurring duplications of *SmE* could be beneficial for plant adaptation, as additional copies could ensure sufficient expression under specific conditions, which might explain a wide range of biotic and abiotic stresses that affect *SmE* expression across different species of the Viridiplantae. Considering the importance of SmE for plant survival, duplications also ensure that at least one functional copy of the gene is always active, while others are available for subfunctionalization [49].

Despite multiple independent duplication events in various clades of the Viridiplantae, the *SmE* CDS and the protein sequences exhibit a very high degree of sequence conservation. This is in line with a study by Chen and Cao 2014 [41], who reported a significant prevalence of synonymous over nonsynonymous mutations during *SmE* evolution. Interestingly, the same study also showed that the exon-intron structure of homologous *Sm* genes was conserved even between distantly related species. In agreement with this report, we observed the same 6 exon/ 5 intron structure found in *AtPCP* in euphyllophytes (ferns and seed plants). In contrast, the representatives of green algae, bryophytes and lycophytes were characterized by single-exon *SmE* coding sequences.

Cao et al. 2011 [43] also reported that duplicated *Sm* genes showed little similarity in their expression patterns despite the high conservation in their intron-exon structure. However, in the case of *AtPCP* and *AtPCPL*, analysis of publicly available expression data indicates their co-expression across a wide range of tissues and treatments. In line with this finding, we observed that the expression of *PCP* and *PCPL* in response to ambient temperatures ranging from 10°C to 27°C is quite similar. Despite these similarities in temperature-dependent gene regulation, we did not observe any temperature-sensitive phenotype in *pcpl* mutants (Fig 4, S3, and S4 Figs).

Interestingly, rice *SmE* orthologs were shown to be induced in response to cold [41,50] reminiscent of *PCP* in *A. thaliana*. Similarly, it has been reported that *SmE* genes in tomato and *Populus* were also differentially expressed in response to cold [51–53]. In contrast, the *SmE* homolog in *C. reinhardtii,* which is encoded by a single-exon gene, was not induced in response to cold [54]. This finding could be interpreted as evidence that expression regulation by cold temperatures is a derived feature and might be conserved in higher land plants. However, considering that gene duplications provide a source for inter- and intraspecies expression and functional diversification [55,56] additional analyses, including the Brassicaceae *SmE* homologs, are required before any conclusions regarding the contribution of the *SmE* gene structure to expression in response to cold can be drawn.

Further investigation of the protein structure of the *A. thaliana* SmE proteins suggested that the two proteins did not functionally diversify. This notion is supported by our finding that substituting the two amino acids that discriminate PCP and PCPL in the context of the full genomic *PCP* rescue construct by either the amino acids found in PCPL or two other small amino acids resulted in the full rescue of the *pcp-1* phenotype at 16 °C (Fig 7). This is in agreement with the observation that gene copies retained after the WGD show lower levels of structural and functional changes in the course of evolution than tandem duplications [57]. Taken together, these findings suggest that the main difference between *PCP* and *PCPL* resides in the differential regulation of the expression of these two genes. In *A. thaliana*, *PCP* might be the more important orthologue, especially at low temperatures, not only due to the differential expression in response to cold but also due to the downstream regulation of the transcripts (Fig 6). As mentioned above, shifts in expression patterns between duplicated genes contribute to the retention of the duplicates [49,58]. Furthermore, the fact that *Brassica rapa* and *Brassica oleracea* have lost their copy of *PCPL* and acquired an additional copy of *PCP* can be interpreted as indirect evidence for the functional redundancy of the two proteins, with PCP being the more important in *A. thaliana.* However, the observed seed abortion rate of 25% in the *pcp-1 pcpl* crossing progeny suggests that plants require at least one functional copy of SmE for survival (Fig 3). It has previously been demonstrated that PCP is important for proper splicing of pre-mRNA [33,34,59,60], but information regarding target preferences of PCPL is still lacking, and we cannot completely exclude the possibility that the two *A. thaliana* SmE proteins interact with different partners or are differentially regulated at some level.

In this context, it is important to note that our results indicate that the expression of *PCP* requires a full genomic sequence including promoter, 5’ UTR, gene region consisting of exons and introns, and downstream sequences. Only such a full genomic construct can rescue the *pcp-1* phenotype at low ambient temperatures. At the same time, expression of the *PCP* cDNA under the control of the *PCP* promoter and terminator cannot (Figs 4A and 5). Expression of the *PCPL* gDNA under the control of the *PCP* promoter and terminator, despite being highly similar to *PCP* in exon-intron structure, was not sufficient to completely rescue the *pcp-1* mutant (Fig 5). It should be noted that Wang et al. 2022 found that expression of a *pPCP::gPCP-3xFLAG* construct lacking the terminator was able to rescue the *pcp-1* phenotype, which suggests that minimal sequence requirements for *PCP* expression do not include the 3’ region. Our intron deletion experiments clearly demonstrate that intronic sequences are required for *PCP* expression and function (Fig 8). Intron 1, in particular, appears to be important as its deletion can lead to complete loss of *PCP* expression. However, the 1^st^ intron on its own (*gPCP-Δi2/3/4/5*) is not sufficient to drive the expression of *PCP* to rescue the *pcp-1* mutant phenotype (Fig 8).

Introns show a very wide range of regulatory mechanisms that affect not only gene expression but also the processing of the transcript, its metabolism, and even translational efficiency, which complicates the study of intron-mediated expression regulation [61,62]. Introns can drive the expression of the gene, affect expression levels and organ-/tissue-specificity, activate transcription, contain *cis*-regulatory elements, provide miRNA-binding sites, and possibly contain elements that act as riboswitches [63–65]. First introns in particular have been shown to play important roles in these regulatory functions, in some cases turning them into the key regulators of gene expression [66–68]. In particular, the phenomenon of intron-mediated enhancement (IME) is strongly linked to the proximity of the given (first) intron to the 5’UTR [69,70].

Interestingly, lines expressing either the full genomic sequence of *PCP* or certain intron-deletion constructs with detectable *PCP* expression also exhibited cold-induced expression (Figs 6 and 8B). These findings suggest that temperature responsiveness is not mediated by these particular intronic sequences. The contribution of intron 1 to the temperature-regulated expression of *PCP* will need to be investigated in more detail as the deletion lines created in this study resulted in varying degrees of *PCP* expression and phenotypic rescue. However, our results nevertheless allow us to conclude that intronic sequences contribute to the cold-responsive expression of *PCP* in a complex and partially redundant manner.

It is also important to note that induction of *PCP* has been reported to be attenuated in a *cbf123* triple mutant background in response to prolonged cold treatment [34], indicating that these key regulators of plant temperature responses contribute to *PCP* regulation. Notably, this regulation appears to be direct as EMSA and ChIP experiments indicated interaction between the promoter region of *PCP* and CBFs at a CRT/DRE *cis*‐regulatory element located approx. 1 kb upstream of the *PCP* start codon [34]. Importantly, the genomic rescue construct used in the current study employed a shorter promoter fragment and lacked the CRT/DRE *cis*-regulatory element. Furthermore, no additional CRT/DRE consensus sequences could be identified in the *gPCP* rescue construct, suggesting that cold responsiveness, in this case, is not mediated by CBFs but rather by an unknown mechanism. It is also worthwhile noting that the CRT/DRE *cis*-regulatory element in the *PCP* upstream region is contained within a recently discovered lncRNA locus, *FLAIL* (At2g18735) [71]. Whether the CRT/DRE element regulates the expression of *FLAIL*, *PCP*, or both remains to be resolved.

In summary, our work provides evidence that SmE is a protein essential for plant growth and development and that the two copies of the SmE protein present in *A. thaliana* perform the same function. However, in contrast to *PCP*, the deletion of *PCPL* does not affect plant growth and development under any of the conditions tested. We attribute these differences to divergence in gene expression as *PCP* is, in general, the more highly expressed of the two genes. Interestingly, our results indicate that intronic sequences are central to *PCP* expression. The molecular mechanism underlying intron-dependent regulation of *PCP* is, however, currently unclear and will need to be investigated further in the future.

## Materials and methods

### Phylogenetic analysis

At2g18740 (AtPCP/AtSmE1) was used to query the Phytozome v13 [72] database to identify potential homologues among the available genomes of Viridiplantae. Species were chosen to represent the major groups of the green plants as best as possible (S1 Table). Genomic DNA, coding DNA, and protein sequences of selected SmE orthologs were downloaded from the NCBI GenBank database (www.ncbi.nlm.nih.gov/genbank/).

The retrieved SmE amino acid sequences of the selected species were aligned using MAFFT v7 (L-INS-i algorithm) [73] and manually checked for mistakes in the alignment using Geneious Prime 2023.0.4 software (https://www.geneious.com/). The corrected amino acid alignment was reverse translated into a corresponding cDNA alignment using Pal2Nal v14 [74]. The output cDNA alignment was used to build a phylogenetic tree using IQ-TREE v2.2.0 [75,76]. The tree was rooted on the green alga *Chlamydomonas reinhardtii.* Command lines used to perform the tree calculation are provided in Supplementary Method S1 Method. Trees were visualized using the iTOL online tool v6 [77].

### Protein structure prediction

Protein domain search and secondary structure prediction were performed on the consensus sequence reconstructed after protein alignment using NCBI Conserved Domains Search [78–80] and Jpred 4 tool [81], respectively.

3D protein structures of PCP (SmE1) and PCPL (SmE2) based on AlphaFold2 predictions [82] were retrieved from the BAR ePlant (https://bar.utoronto.ca/eplant/) [83]. Protein structures were visualized using PyMOL, version 3.0.2 (Schrödinger, LLC).

### Plant material and growth conditions

All work was done using *Arabidopsis thaliana* plants of Col-0 accession. The *pcp-1* T-DNA insertion line (SALK_089521) has previously been described [32]. The *pcpl-1* and *pcpl-2* deletion mutants were generated as part of this study. Briefly, *A. thaliana* Col-0 plants were transformed by floral dipping with constructs for CRISPR/Cas9-mediated mutagenesis (described below) [84]. Primary transformants (T1) carrying deletions in *PCPL* were identified, and homozygous transgene-free *pcpl-1* mutants were isolated in subsequent T2/T3 generations.

Seeds were stratified at 4°C in the dark for 48 h before sowing on soil or plates. Seeds grown in sterile conditions were sown on ½  MS media [85] plates with 1.6% agar. Plates for the selection of transformants were additionally supplemented with 10 ug/mL phosphinotricin (Duchefa Biochemie) (BASTA). Seeds sown on plates were surface sterilized beforehand with 0.015% Triton X-100 solution in 70% EtOH for 10 minutes, washed once with 96% EtOH and three times with MQ water.

Plants were grown in long day (LD) conditions (16 h light/8h dark) in Percival chambers equipped with full range LED illumination with the following conditions: light intensity of 120 μmol * m^-2^s^-1^, constant humidity (RH 65%) at either 10°C, 16°C, 23°C, or 27°C.

### Plant transformation and selection of transformants

Electro-competent *Agrobacterium tumefaciens* GV3101 cells containing the pSOUP plasmid required for the replication of pGreen-IIS-based vectors [86,87] were transformed with the plasmids described below and grown on selective media using the appropriate antibiotics. *A. thaliana pcp-1* mutants were grown at 23°C till flowering stage before siliques started to develop. Plants were transformed by floral dipping using *Agrobacterium tumefaciens*-mediated gene transfer [84]. T1 seeds were selected by growing on ½  MS media plates with 1.6% agar supplemented with 10 ug/mL BASTA. Seedlings exhibiting BASTA-resistant phenotype were transferred to soil and the presence of insertion or deletion was confirmed by PCR (S4 Table). Alternatively, the selection of transformants expressing the mCherry or GFP seed marker was performed by picking T1 fluorescent-positive seeds using an epifluorescent stereomicroscope.

To remove the CRISPR/Cas9 cassette, plants were backcrossed to Col-0 and transgene-free plants with deletions in *PCPL* were established (S1 Fig).

### Plant phenotyping

Phenotypes are reported for homozygous mutants and transgenic lines for which segregation analysis indicated the presence of a single T-DNA insertion unless otherwise stated. Phenotypic analyses were performed using plants grown on ½  MS plates at 10°C, 16°C, 23°C, and 27°C for 25, 10, 10, and 7 days, respectively. Rescue assays of *pcp-1* plants transformed with *PCP* intron deletions were performed in seedlings grown on ½  MS plates at 16°C for 10 days. Plates for root growth assays were scanned using an EPSON Expression 12000XL scanner equipped with SilverFast^®^ 8 software at 1800 dpi resolution or photographed. In addition, pictures were taken using a digital camera to document rosette phenotypes.

Embryo lethality in self-pollinated *pcp-1*/*pcpl-1* (het/het), *pcp-1*/*pcpl-2* (het/het) lines was calculated as the proportion of aborted embryos relative to the total amount of healthy seeds and aborted embryos on one side of the silique.

### Cloning of DNA fragments and assembly of plasmids

Genomic *PCP* and *PCPL* fragments were amplified by PCR using primers listed in S4 Table from DNA extracted from Col-0 using the protocol described by [88] with the following modifications: the volume of the extraction buffer added to the sample before the grinding step was reduced to 500 μl and the incubation time at 95°C was reduced to 1 minute. Intron deletion constructs in *PCP* were created by overlap extension PCR [89] using the primers listed in S4 Table. *PCP* and *PCPL* open reading frames (ORFs) were amplified from cDNA. For this, total RNA was isolated from Col-0 seedlings using the RNeasy^®^ Plant Mini Kit (Qiagen) following the manufacturer’s protocol. Purified RNA was treated with DNase I (Thermo Scientific) followed by first-strand cDNA synthesis using the RevertAid RT kit (Thermo Scientific) with oligo(dT)_18_ primer. The primers used for amplification of *PCP* and *PCPL* Coding Sequences (CDSs) by PCR are listed in S4 Table.

*PCP* and *PCPL* genomic sequences and ORFs were cloned into GreenGate C-module entry plasmids as previously described [87]. *PCP* promoter (232 bp upstream of the start codon, covering the 5’-UTR and intergenic region) and *PCP* terminator (423 bp downstream the stop codon, including the 3’-UTR and intergenic region) sequences were amplified from Col-0 DNA using primers listed in S4 Table and cloned into GreenGate entry vectors A and E, respectively. For the generation of *PCP* and *PCPL* overexpression lines, existing A- and E-modules carrying 35S promoter and pea RbcS terminator sequences, respectively, were used [87].

The plant binary plasmids were assembled in destination vector pGGZ003 using GreenGate cloning as previously described [87]. Empty B- and D-modules were used in the assembly reaction. BASTA resistance was used as a selection marker in module F in all constructs except *PCP* intron deletion lines, in which case a seed-specific mCherry selection marker was used [90–92].

Point mutations to exchange amino acids in positions 65 and 86 in PCP and PCPL were generated by PCR using assembled plasmids with *gPCP* (pVD_007) and *gPCPL* (pVD_008) insertions as a template (S5 Table) and primers listed in S4 Table.

The final plasmids assembled by GreenGate cloning are listed in S2 Table. The remaining plasmids were generated by amplifying the inserts using overlap fusion PCR to simultaneously introduce the desired mutations/deletions and A- and G-compatible GreenGate sites, followed by GreenGate cloning into the pGGZ003 destination (S3 Table).

For CRISPR/Cas9-mutagenesis of *PCPL*, pairs of gene-specific sgRNAs were designed using CRISPR-P 2.0 (http://crispr.hzau.edu.cn/CRISPR2/) to simultaneously target two positions in the gene (S1 Fig). Selected pairs of sgRNAs were introduced into forward and reverse primers (S1 Fig and S4 Table), which were used to amplify a dual-guideRNA cassette via the pCBC-DT1T2 vector [93] thereby introducing a sgRNA scaffold to the first guide RNA sequence, a U6-29 Promoter to the second guide RNA sequence and flanking BsaI restriction sites, making it compatible with GreenGate-based CRISPR vectors. The PCR amplicons were purified using the E.Z.N.A.^®^ Gel Extraction Kit (Omega Bio-tek) and cloned into BsaI-compatible sites of the vector CF588 via the GreenGate reaction.

All constructs were transformed and propagated in DH5α *Escherichia coli* cells. Single colonies were selected on solid LB media supplemented with the appropriate antibiotic, propagated in liquid LB media, and plasmids were purified using the E.Z.N.A.^®^ Plasmid DNA Mini Kit I (Omega Bio-tek). All plasmids generated in this study were verified by Sanger sequencing using insert-specific primers (S4 Table) and are listed in S2 and S3 Tables.

All primers used in this study were synthesized by Eurofins Genomics.

### RNA extraction and RT-qPCR analysis

For RT-qPCR plants were grown on ½  MS media plates with 1.6% agar at 23°C for 5 days and then transferred to the temperature of interest (10°C, 16°C, 23°C and 27°C) for one more day of growth. Approximately 10 seedlings per biological replicate were collected in Eppendorf tubes^®^, immediately flash-frozen in liquid nitrogen, and stored at -80°C. The frozen plant material was ground to fine powder using a Mixer Mill MM 400 (Retsch) and stainless-steel beads. All steps were performed with cooled-down equipment to prevent premature thawing of the samples. Extraction of RNA was performed using the RNeasy^®^ Plant Mini Kit (Qiagen) following manufacturer’s protocol. RNA concentration and quality were measured using a NanoDrop™ 2000 Spectrophotometer (Thermo Scientific) device and RNA was stored at -80°C till further use.

Reverse transcription was carried out from 1 μg of purified RNA using the RevertAid RT kit (Thermo Scientific) with oligo(dT)_18_ primer. Single-strand cDNA was stored at -20°C. RT-qPCR reactions were prepared using the LightCycler® 480 SYBR Green I Master mix. All RT-qPCR assays were performed on CFX™ Real-Time PCR Detection System (Bio-Rad, Inc., Hercules, CA, USA) instruments equipped with either 96- or 384-well blocks using the following program: 5 min at 95°C, followed by 40 cycles of 15 sec at 95°C (denaturation), 30 sec at 55°C (annealing), 20 sec at 72°C (elongation), followed by a melting curve analysis from 55°C to 95°C using 1°C increments. Relative gene expression was calculated using the 2^–^^△△^^Ct^ method. with *TUB2* (AT5G62690) as a reference gene. All RT-qPCR assays were performed using 3 biological replicates with 3 technical repetitions each except lines expressing pNR_060_5-2, pNR_121_4-1-6, pNR_157_1-2 constructs that lacked material for 3 biological replicates. All primers used for RT-qPCR analyses can be found in the S6 Table.

### Image processing

Figures were processed and prepared for publication using Affinity Designer v1.10.6 (Serif Ltd, https://affinity.serif.com/en-us/).

### Statistical analysis

Root length was calculated using Fiji 2.14.0 (ImageJ) [94]. Calculation of the average root length, one-way ANOVA with Tukey’s test to estimate the difference between genotypes and data plotting was performed with the GraphPad Prism version 10.2.2 for Windows, GraphPad Software, Boston, Massachusetts USA, www.graphpad.com. Similarly, calculation of the average, Tukey test to eliminate the outliers and data plotting was performed for abortion rates in the mutants.

## Supporting information

S1 Method Command line instructions used for phylogenetic analysis. (DOCX)

S1 Fig *Arabidopsis thaliana* SmE protein structure. Overlay of PCP (SmE1) (teal) and PCPL (SmE2) (orange) illustrates the high similarity of the predicted structures. N- and C-terminals of the proteins and amino acids in positions 65 and 86 are marked on the protein structures with the corresponding colors.
(TIF)

S2 Fig CRISPR/Cas9-generated *PCPL* mutant alleles. Shown are the region of the genomic *PCPL* sequence (*PCPL* gDNA) into which a mutation (deletion) was introduced. Boxes mark exon sequence and dashes indicate the deleted nucleotides. Lines above the gDNA indicate the position of sgRNAs. Underlined letters mark the in-frame stop codons generated in the truncated *PCPL* coding sequence (CDS). The predicted protein sequence is shown at the bottom with bold letters indicating the first altered amino acid.
(TIF)

S3 Fig Plant phenotypes at 27°C and 10°C. Representative images of the 7-day-old seedlings transformed with various rescue constructs grown at 27°C (A) and 25-day-old seedlings grown at 10°C (B). Three independent lines are shown for each rescue construct generated in this study: #1-3: *pPCP::cPCP::tPCP*; #4-6: *pPCP::cPCPL::tPCP*; #7-9: *pPCP::gPCP::tPCP*; #10-12: *pPCP::gPCPL::tPCP*; #13: *35S::cPCP::tRbcS* [32]; #14-16: *35S::cPCPL::tRbcS*. All constructs were expressed in the *pcp-1* background.
(TIF)

S4 Fig Plant phenotypes at 23°C and 16°C. Representative images of the 10-day-old seedlings transformed with various rescue constructs grown at 23°C (A) and 16°C (B). Three independent lines are shown for each rescue construct generated in this study: #1-3: *pPCP::cPCP::tPCP*; #4-6: *pPCP::cPCPL::tPCP*; #7-9: *pPCP::gPCP::tPCP*; #10-12: *pPCP::gPCPL::tPCP*; #13: *35S::cPCP::tRbcS* [32]; #14-16: *35S::cPCPL::tRbcS*. All constructs were expressed in the *pcp-1* background.
(TIF)

S5 Fig Root length of *PCP* intron-deletion lines. Length of the primary root of three independent lines in the *pcp-1* background transformed with various intron deletion constructs. Deletion lines and constructs are labeled as *gPCP_ ∆ iX*, where *gPCP* represents a full *pPCP::gPCP::tPCP* construct, ∆  stands for deletion, i stands for intron, and X indicates the number(s) of the deleted intron(s). Col-0, *pcp-1*, and a *pcp-1* rescue line carrying the full-length *pPCP::gPCP::tPCP* construct serve as controls. Root length was determined using 10-day-old seedlings grown at 16°C (blue) and 23°C (light grey) for three independent transgenic lines per intron-deletion construct. Bars show the mean of the root length measurement (n =  10 to 37), and error bars indicate the standard deviation (SD). A and B show independent measurements. A two-way ANOVA test with Tukey correction was performed using GraphPad Prism. Letters represent significantly different (P <  0.05) groups.
(TIF)

S6 Fig *PCP* expression and root phenotype rescue in independent *gPCP_* ∆ *i1* lines. A) Difference in the length of the primary root of independent *gPCP_ ∆ i1* lines in the *pcp-1* background. Col-0 and *pcp-1* serve as controls. Root length was determined using 10-day-old seedlings grown at 16°C (blue) and 23°C (light grey). Bars show the mean of the root length measurement (n =  10 to 28), and error bars indicate the standard deviation (SD). B) Corresponding expression of *PCP*. Gene expression was determined in seedlings after 5 days of growth at 23°C followed by 24 h of growth at 16°C (blue) or 23°C (grey). Bars show the mean expression calculated from three biological replicates with three technical replicates each. Error bars indicate the standard deviation (SD). A two-way ANOVA test with Tukey correction was performed using GraphPad Prism. Letters represent significantly different (P <  0.05) groups.
(TIF)

S1 Table *SmE* genes used in phylogenetic analysis. (DOCX)

S2 Table GreenGate plant binary plasmids used in this study. (DOCX)

S3 Table Summary of plasmids cloned by overlap PCR used in this study. (DOCX)

S4 Table Oligonucleotides used in this study. (DOCX)

S5 Table Summary of GreenGate entry modules generated in this study, including information on PCR primers used for amplification. (DOCX)

S6 Table Primers used for RT-qPCR. (DOCX)

## References

[1] Quint M, Delker C, Franklin KA, Wigge PA, Halliday KJ, van Zanten M. Molecular and genetic control of plant thermomorphogenesis. Nat Plants. 2016;2:15190. doi: 10.1038/nplants.2015.190 27250752

[2] Hasanuzzaman M, Nahar K, Alam MM, Roychowdhury R, Fujita M. Physiological, biochemical, and molecular mechanisms of heat stress tolerance in plants. Int J Mol Sci. 2013;14(5):9643–84. doi: 10.3390/ijms14059643 23644891 PMC3676804

[3] Chinnusamy V, Zhu J, Zhu J-K. Cold stress regulation of gene expression in plants. Trends Plant Sci. 2007;12(10):444–51. doi: 10.1016/j.tplants.2007.07.002 17855156

[4] Baier M, Bittner A, Prescher A, van Buer J. Preparing plants for improved cold tolerance by priming. Plant Cell Environ. 2019;42(3):782–800. doi: 10.1111/pce.13394 29974962

[5] Graham D, Patterson BD. Responses of plants to low, nonfreezing temperatures: Proteins, metabolism, and acclimation. Annu Rev Plant Physiol. 1982;33(1):347–72. doi: 10.1146/annurev.pp.33.060182.002023

[6] Guy CL. Cold acclimation and freezing stress tolerance: Role of protein metabolism. Annu Rev Plant Physiol Plant Mol Biol. 1990;41(1):187–223. doi: 10.1146/annurev.pp.41.060190.001155

[7] Orvar BL, Sangwan V, Omann F, Dhindsa RS. Early steps in cold sensing by plant cells: The role of actin cytoskeleton and membrane fluidity. Plant J. 2000;23(6):785–94. doi: 10.1046/j.1365-313x.2000.00845.x 10998189

[8] Ruelland E, Zachowski A. How plants sense temperature. Environ Exp Bot. 2010;69(3):225–32. doi: 10.1016/j.envexpbot.2010.05.011

[9] Miura K, Furumoto T. Cold signaling and cold response in plants. Int J Mol Sci. 2013;14(3):5312–37. doi: 10.3390/ijms14035312 23466881 PMC3634503

[10] Lamers J, van der Meer T, Testerink C. How plants sense and respond to stressful environments. Plant Physiol. 2020;182(4):1624–35. doi: 10.1104/pp.19.01464 32132112 PMC7140927

[11] Manasa S L, Panigrahy M, Panigrahi KCS, Rout GR. Overview of cold stress regulation in plants. Bot Rev. 2021;88(3):359–87. doi: 10.1007/s12229-021-09267-x

[12] Shi Y, Ding Y, Yang S. Cold signal transduction and its interplay with phytohormones during cold acclimation. Plant Cell Physiol. 2015;56(1):7–15. doi: 10.1093/pcp/pcu115 25189343

[13] Berget SM, Moore C, Sharp PA. Spliced segments at the 5’ terminus of adenovirus 2 late mRNA. Proc Natl Acad Sci U S A. 1977;74(8):3171–5. doi: 10.1073/pnas.74.8.3171 269380 PMC431482

[14] Gilbert W. Why genes in pieces? Nature. 1978;271(5645):501. doi: 10.1038/271501a0 622185

[15] Syed NH, Kalyna M, Marquez Y, Barta A, Brown JWS. Alternative splicing in plants--coming of age. Trends Plant Sci. 2012;17(10):616–23. doi: 10.1016/j.tplants.2012.06.001 22743067 PMC3466422

[16] Staiger D, Brown JWS. Alternative splicing at the intersection of biological timing, development, and stress responses. Plant Cell. 2013;25(10):3640–56. doi: 10.1105/tpc.113.113803 24179132 PMC3877812

[17] Shang X, Cao Y, Ma L. Alternative splicing in plant genes: A means of regulating the environmental fitness of plants. Int J Mol Sci. 2017;18(2):432. doi: 10.3390/ijms18020432 28230724 PMC5343966

[18] Liu X-X, Guo Q-H, Xu W-B, Liu P, Yan K. Rapid regulation of alternative splicing in response to environmental stresses. Front Plant Sci. 2022;13:832177. doi: 10.3389/fpls.2022.832177 35310672 PMC8931528

[19] Kelemen O, Convertini P, Zhang Z, Wen Y, Shen M, Falaleeva M, et al. Function of alternative splicing. Gene. 2013;514(1):1–30. doi: 10.1016/j.gene.2012.07.083 22909801 PMC5632952

[20] Reddy ASN, Marquez Y, Kalyna M, Barta A. Complexity of the alternative splicing landscape in plants. Plant Cell. 2013;25(10):3657–83. doi: 10.1105/tpc.113.117523 24179125 PMC3877793

[21] Filichkin SA, Priest HD, Givan SA, Shen R, Bryant DW, Fox SE, et al. Genome-wide mapping of alternative splicing in Arabidopsis thaliana. Genome Res. 2010;20(1):45–58. doi: 10.1101/gr.093302.109 19858364 PMC2798830

[22] Marquez Y, Brown JWS, Simpson C, Barta A, Kalyna M. Transcriptome survey reveals increased complexity of the alternative splicing landscape in Arabidopsis. Genome Res. 2012;22(6):1184–95. doi: 10.1101/gr.134106.111 22391557 PMC3371709

[23] Zhang R, Kuo R, Coulter M, Calixto CPG, Entizne JC, Guo W, et al. A high-resolution single-molecule sequencing-based Arabidopsis transcriptome using novel methods of Iso-seq analysis. Genome Biol. 2022;23(1):149. doi: 10.1186/s13059-022-02711-0 35799267 PMC9264592

[24] Szakonyi D, Duque P. Alternative splicing as a regulator of early plant development. Front Plant Sci. 2018;9:1174. doi: 10.3389/fpls.2018.01174 30158945 PMC6104592

[25] Laloum T, Martín G, Duque P. Alternative splicing control of abiotic stress responses. Trends Plant Sci. 2018;23(2):140–50. doi: 10.1016/j.tplants.2017.09.019 29074233

[26] Tognacca RS, Rodríguez FS, Aballay FE, Cartagena CM, Servi L, Petrillo E. Alternative splicing in plants: Current knowledge and future directions for assessing the biological relevance of splice variants. J Exp Bot. 2023;74(7):2251–72. doi: 10.1093/jxb/erac431 36306285

[27] Calixto CPG, Guo W, James AB, Tzioutziou NA, Entizne JC, Panter PE, et al. Rapid and dynamic alternative splicing impacts the Arabidopsis cold response transcriptome. Plant Cell. 2018;30(7):1424–44. doi: 10.1105/tpc.18.00177 29764987 PMC6096597

[28] Jiang J, Liu X, Liu C, Liu G, Li S, Wang L. Integrating omics and alternative splicing reveals insights into grape response to high temperature. Plant Physiol. 2017;173(2):1502–18. doi: 10.1104/pp.16.01305 28049741 PMC5291026

[29] Li Y, Mi X, Zhao S, Zhu J, Guo R, Xia X, et al. Comprehensive profiling of alternative splicing landscape during cold acclimation in tea plant. BMC Genomics. 2020;21(1):65. doi: 10.1186/s12864-020-6491-6 31959105 PMC6971990

[30] Zhong Y, Luo Y, Sun J, Qin X, Gan P, Zhou Z, et al. Pan-transcriptomic analysis reveals alternative splicing control of cold tolerance in rice. Plant Cell. 2024;36(6):2117–39. doi: 10.1093/plcell/koae039 38345423 PMC11132889

[31] Dikaya V, El Arbi N, Rojas-Murcia N, Nardeli SM, Goretti D, Schmid M. Insights into the role of alternative splicing in plant temperature response. J Exp Bot. 2021;72 (21):7384–403. doi: 10.1093/jxb/erab234 34105719

[32] Capovilla G, Delhomme N, Collani S, Shutava I, Bezrukov I, Symeonidi E, et al. PORCUPINE regulates development in response to temperature through alternative splicing. Nat Plants. 2018;4(8):534–9. doi: 10.1038/s41477-018-0176-z 29988152

[33] Huertas R, Catalá R, Jiménez-Gómez JM, Mar Castellano M, Crevillén P, Piñeiro M, et al. Arabidopsis SME1 regulates plant development and response to abiotic stress by determining spliceosome activity specificity. Plant Cell. 2019;31(2):537–54. doi: 10.1105/tpc.18.00689 30696706 PMC6447010

[34] Wang Z, Hong Y, Yao J, Huang H, Qian B, Liu X, et al. Modulation of plant development and chilling stress responses by alternative splicing events under control of the spliceosome protein SmEb in Arabidopsis. Plant Cell Environ. 2022;45(9):2762–79. doi: 10.1111/pce.14386 35770732

[35] Khusial P, Plaag R, Zieve GW. LSm proteins form heptameric rings that bind to RNA via repeating motifs. Trends Biochem Sci. 2005;30(9):522–8. doi: 10.1016/j.tibs.2005.07.006 16051491

[36] Will CL, Lührmann R. Spliceosome structure and function. Cold Spring Harb Perspect Biol. 2011;3(7):a003707. doi: 10.1101/cshperspect.a003707 21441581 PMC3119917

[37] Hermann H, Fabrizio P, Raker VA, Foulaki K, Hornig H, Brahms H, et al. snRNP Sm proteins share two evolutionarily conserved sequence motifs which are involved in Sm protein-protein interactions. EMBO J. 1995;14(9):2076–88. doi: 10.1002/j.1460-2075.1995.tb07199.x 7744013 PMC398308

[38] Kambach C, Walke S, Young R, Avis JM, de la Fortelle E, Raker VA, et al. Crystal structures of two Sm protein complexes and their implications for the assembly of the spliceosomal snRNPs. Cell. 1999;96:375–87. doi: 10.1016/s0092-8674(00)80550-4 10025403

[39] Weber G, Trowitzsch S, Kastner B, Lührmann R, Wahl MC. Functional organization of the Sm core in the crystal structure of human U1 snRNP. EMBO J. 2010;29:4172–84. doi: 10.1038/emboj.2010.295 21113136 PMC3018796

[40] El Arbi N, Nardeli SM, Šimura J, Ljung K, Schmid M. The Arabidopsis splicing factor PORCUPINE/SmE1 orchestrates temperature‐dependent root development via auxin homeostasis maintenance. New Phytologist. 2024;244(4):1408–21. doi: 10.1111/nph.20153 39327913

[41] Chen Y, Cao J. Comparative genomic analysis of the Sm gene family in rice and maize. Gene. 2014;539(2):238–49. doi: 10.1016/j.gene.2014.02.006 24525402

[42] Gao R, Su N, Pan W, Bao Q, Li Z, Nie X, et al. Genome-wide investigation of spliceosomal SM/LSM genes in wheat (Triticum aestivum L.) and its progenitors. Agronomy. 2021;11. doi: 10.3390/agronomy11071429

[43] Cao J, Shi F, Liu X, Jia J, Zeng J, Huang G. Genome-wide identification and evolutionary analysis of Arabidopsis sm genes family. J Biomol Struct Dyn. 2011;28:535–44. doi: 10.1080/07391102.2011.10508593 21142222

[44] Zuntini AR, Carruthers T, Maurin O, Bailey PC, Leempoel K, Brewer GE, et al. Phylogenomics and the rise of the angiosperms. Nature. 2024;629:843–50. doi: 10.1038/s41586-024-07324-0 38658746 PMC11111409

[45] Thompson JD, Gibson TJ, Plewniak F, Jeanmougin F, Higgins DG. The CLUSTAL_X windows interface: Flexible strategies for multiple sequence alignment aided by quality analysis tools. Nucleic Acids Res. 1997;25:4876–82. doi: 10.1093/nar/25.24.4876 9396791 PMC147148

[46] Kiselev KV, Aleynova OA, Ogneva ZV, Suprun AR, Dubrovina AS. 35S promoter-driven transgenes are variably expressed in different organs of Arabidopsis thaliana and in response to abiotic stress. Mol Biol Rep. 2021;48:2235–41. doi: 10.1007/s11033-021-06235-x 33630207

[47] Flagel LE, Wendel JF. Gene duplication and evolutionary novelty in plants. New Phytol. 2009;183(3):557–64. doi: 10.1111/j.1469-8137.2009.02923.x 19555435

[48] Barker MS, Vogel H, Schranz ME. Paleopolyploidy in the Brassicales: analyses of the Cleome transcriptome elucidate the history of genome duplications in Arabidopsis and other Brassicales. Genome Biol Evol. 2009;1:391–9. doi: 10.1093/gbe/evp040 20333207 PMC2817432

[49] Blanc G, Wolfe KH. Functional divergence of duplicated genes formed by polyploidy during Arabidopsis evolution. Plant Cell. 2004;16(7):1679–91. doi: 10.1105/tpc.021410 15208398 PMC514153

[50] Kawahara Y, Oono Y, Wakimoto H, Ogata J, Kanamori H, Sasaki H, et al. TENOR: Database for comprehensive mRNA-Seq experiments in rice. Plant Cell Physiol. 2016;57:1–13. doi: 10.1093/pcp/pcv179 26578693

[51] Chen H, Chen X, Chen D, Li J, Zhang Y, Wang A. A comparison of the low temperature transcriptomes of two tomato genotypes that differ in freezing tolerance: Solanum lycopersicum and Solanum habrochaites. BMC Plant Biol. 2015;15:132. doi: 10.1186/s12870-015-0521-6 26048292 PMC4458020

[52] Barrero-Gil J, Huertas R, Rambla JL, Granell A, Salinas J. Tomato plants increase their tolerance to low temperature in a chilling acclimation process entailing comprehensive transcriptional and metabolic adjustments. Plant Cell Environ. 2016;39(10):2303–18. doi: 10.1111/pce.12799 27411783

[53] Filichkin SA, Hamilton M, Dharmawardhana PD, Singh SK, Sullivan C, Ben-Hur A, et al. Abiotic stresses modulate landscape of poplar transcriptome via alternative splicing, differential intron retention, and isoform ratio switching. Front Plant Sci. 2018;9:5. doi: 10.3389/fpls.2018.00005 29483921 PMC5816337

[54] Li L, Peng H, Tan S, Zhou J, Fang Z, Hu Z, et al. Effects of early cold stress on gene expression in Chlamydomonas reinhardtii. Genomics. 2020;112(2):1128–38. doi: 10.1016/j.ygeno.2019.06.027 31251979

[55] Gu Z, Rifkin SA, White KP, Li W-H. Duplicate genes increase gene expression diversity within and between species. Nat Genet. 2004;36(6):577–9. doi: 10.1038/ng1355 15122255

[56] Ha M, Kim E-D, Chen ZJ. Duplicate genes increase expression diversity in closely related species and allopolyploids. Proc Natl Acad Sci U S A. 2009;106(7):2295–300. doi: 10.1073/pnas.0807350106 19168631 PMC2650150

[57] Wang Y, Tan X, Paterson AH. Different patterns of gene structure divergence following gene duplication in Arabidopsis. BMC Genomics. 2013;14:652. doi: 10.1186/1471-2164-14-652 24063813 PMC3848917

[58] Duarte JM, Cui L, Wall PK, Zhang Q, Zhang X, Leebens-Mack J, et al. Expression pattern shifts following duplication indicative of subfunctionalization and neofunctionalization in regulatory genes of Arabidopsis. Mol Biol Evol. 2006;23(2):469–78. doi: 10.1093/molbev/msj051 16280546

[59] Hong Y, Yao J, Shi H, Chen Y, Zhu J-K, Wang Z. The Arabidopsis spliceosomal protein SmEb modulates ABA responses by maintaining proper alternative splicing of HAB1. Stress Biol. 2021;1(1):4. doi: 10.1007/s44154-021-00006-1 37676319 PMC10441929

[60] Hong Y, Gao Y, Pang J, Shi H, Li T, Meng H, et al. The Sm core protein SmEb regulates salt stress responses through maintaining proper splicing of RCD1 pre-mRNA in Arabidopsis. J Integr Plant Biol. 2023;65(6):1383–93. doi: 10.1111/jipb.13457 36661041

[61] Le Hir H, Nott A, Moore MJ. How introns influence and enhance eukaryotic gene expression. Trends Biochem Sci. 2003;28(4):215–20. doi: 10.1016/S0968-0004(03)00052-5 12713906

[62] Chorev M, Carmel L. The function of introns. Front Genet. 2012;3:55. doi: 10.3389/fgene.2012.00055 22518112 PMC3325483

[63] XIE X. Introns in higher plant genes. Chinese Sci Bull. 2002;47(17):1409. doi: 10.1360/02tb9311

[64] Meng Y, Shao C, Ma X, Wang H. Introns targeted by plant microRNAs: A possible novel mechanism of gene regulation. Rice (N Y). 2013;6(1):8. doi: 10.1186/1939-8433-6-8 24280590 PMC4883735

[65] Meng F, Zhao H, Zhu B, Zhang T, Yang M, Li Y, et al. Genomic editing of intronic enhancers unveils their role in fine-tuning tissue-specific gene expression in Arabidopsis thaliana. Plant Cell. 2021;33(6):1997–2014. doi: 10.1093/plcell/koab093 33764459 PMC8290289

[66] Salgueiro S, Pignocchi C, Parry MA. Intron-mediated gusA expression in tritordeum and wheat resulting from particle bombardment. Plant Mol Biol. 2000;42(4):615–22. doi: 10.1023/a:1006331831858 10809007

[67] Zalabák D, Ikeda Y. First come, first served: Sui generis features of the first intron. Plants (Basel). 2020;9(7):911. doi: 10.3390/plants9070911 32707681 PMC7411622

[68] Zhu X, Chen A, Butler NM, Zeng Z, Xin H, Wang L, et al. Molecular dissection of an intronic enhancer governing cold-induced expression of the vacuolar invertase gene in potato. Plant Cell. 2024;36(5):1985–99. doi: 10.1093/plcell/koae050 38374801 PMC11062429

[69] Rose AB. The effect of intron location on intron-mediated enhancement of gene expression in Arabidopsis. Plant J. 2004;40(5):744–51. doi: 10.1111/j.1365-313X.2004.02247.x 15546357

[70] Gallegos JE, Rose AB. The enduring mystery of intron-mediated enhancement. Plant Sci. 2015;237:8–15. doi: 10.1016/j.plantsci.2015.04.017 26089147

[71] Jin Y, Ivanov M, Dittrich AN, Nelson AD, Marquardt S. LncRNA FLAIL affects alternative splicing and represses flowering in Arabidopsis. EMBO J. 2023;42(11):e110921. doi: 10.15252/embj.2022110921 37051749 PMC10233378

[72] Goodstein DM, Shu S, Howson R, Neupane R, Hayes RD, Fazo J, et al. Phytozome: A comparative platform for green plant genomics. Nucleic Acids Res. 2012;40. doi: 10.1093/nar/gkr944 22110026 PMC3245001

[73] Katoh K, Standley DM. MAFFT multiple sequence alignment software version 7: Improvements in performance and usability. Mol Biol Evol. 2013;30(4):772–80. doi: 10.1093/molbev/mst010 23329690 PMC3603318

[74] Suyama M, Torrents D, Bork P. PAL2NAL: Robust conversion of protein sequence alignments into the corresponding codon alignments. Nucleic Acids Res. 2006;34(Web Server issue):W609-12. doi: 10.1093/nar/gkl315 16845082 PMC1538804

[75] Kalyaanamoorthy S, Minh BQ, Wong TKF, von Haeseler A, Jermiin LS. ModelFinder: Fast model selection for accurate phylogenetic estimates. Nat Methods. 2017;14(6):587–9. doi: 10.1038/nmeth.4285 28481363 PMC5453245

[76] Minh BQ, Schmidt HA, Chernomor O, Schrempf D, Woodhams MD, von Haeseler A, et al. IQ-TREE 2: New models and efficient methods for phylogenetic inference in the genomic era. Mol Biol Evol. 2020;37(5):1530–4. doi: 10.1093/molbev/msaa015 32011700 PMC7182206

[77] Letunic I, Bork P. Interactive Tree Of Life (iTOL): An online tool for phylogenetic tree display and annotation. Bioinformatics. 2007;23(1):127–8. doi: 10.1093/bioinformatics/btl529 17050570

[78] Marchler-Bauer A, Bo Y, Han L, He J, Lanczycki CJ, Lu S, et al. CDD/SPARCLE: Functional classification of proteins via subfamily domain architectures. Nucleic Acids Res. 2017;45(D1):D200–3. doi: 10.1093/nar/gkw1129 27899674 PMC5210587

[79] Lu S, Wang J, Chitsaz F, Derbyshire MK, Geer RC, Gonzales NR, et al. CDD/SPARCLE: The conserved domain database in 2020. Nucleic Acids Res. 2020;48(D1):D265–8. doi: 10.1093/nar/gkz991 31777944 PMC6943070

[80] Wang J, Chitsaz F, Derbyshire MK, Gonzales NR, Gwadz M, Lu S, et al. The conserved domain database in 2023. Nucleic Acids Res. 2023;51(D1):D384–8. doi: 10.1093/nar/gkac1096 36477806 PMC9825596

[81] Drozdetskiy A, Cole C, Procter J, Barton GJ. JPred4: A protein secondary structure prediction server. Nucleic Acids Res. 2015;43(W1):W389-94. doi: 10.1093/nar/gkv332 25883141 PMC4489285

[82] Jumper J, Evans R, Pritzel A, Green T, Figurnov M, Ronneberger O, et al. Highly accurate protein structure prediction with AlphaFold. Nature. 2021;596(7873):583–9. doi: 10.1038/s41586-021-03819-2 34265844 PMC8371605

[83] Waese J, Fan J, Pasha A, Yu H, Fucile G, Shi R, et al. ePlant: Visualizing and exploring multiple levels of data for hypothesis generation in plant biology. Plant Cell. 2017;29(8):1806–21. doi: 10.1105/tpc.17.00073 28808136 PMC5590499

[84] Clough SJ, Bent AF. Floral dip: A simplified method for Agrobacterium-mediated transformation of Arabidopsis thaliana. Plant J. 1998;16(6):735–43. doi: 10.1046/j.1365-313x.1998.00343.x 10069079

[85] Murashige T, Skoog F. A revised medium for rapid growth and bio assays with tobacco tissue cultures. Physiol Plant. 1962;15(3):473–97. doi: 10.1111/j.1399-3054.1962.tb08052.x

[86] Hellens RP, Edwards EA, Leyland NR, Bean S, Mullineaux PM. pGreen: A versatile and flexible binary Ti vector for Agrobacterium-mediated plant transformation. Plant Mol Biol. 2000;42(6):819–32. doi: 10.1023/a:1006496308160 10890530

[87] Lampropoulos A, Sutikovic Z, Wenzl C, Maegele I, Lohmann JU, Forner J. GreenGate---a novel, versatile, and efficient cloning system for plant transgenesis. PLoS One. 2013;8(12):e83043. doi: 10.1371/journal.pone.0083043 24376629 PMC3869738

[88] Berendzen K, Searle I, Ravenscroft D, Koncz C, Batschauer A, Coupland G, et al. A rapid and versatile combined DNA/RNA extraction protocol and its application to the analysis of a novel DNA marker set polymorphic between Arabidopsis thaliana ecotypes Col-0 and Landsberg erecta. Plant Methods. 2005;1(1):4. doi: 10.1186/1746-4811-1-4 16270938 PMC1277017

[89] Horton RM, Hunt HD, Ho SN, Pullen JK, Pease LR. Engineering hybrid genes without the use of restriction enzymes: Gene splicing by overlap extension. Gene. 1989;77(1):61–8. doi: 10.1016/0378-1119(89)90359-4 2744488

[90] Gao X, Chen J, Dai X, Zhang D, Zhao Y. An effective strategy for reliably isolating heritable and Cas9-Free Arabidopsis mutants generated by CRISPR/Cas9-Mediated genome editing. Plant Physiol. 2016;171(3):1794–800. doi: 10.1104/pp.16.00663 27208253 PMC4936589

[91] Capovilla G, Symeonidi E, Wu R, Schmid M. Contribution of major FLM isoforms to temperature-dependent flowering in Arabidopsis thaliana. J Exp Bot. 2017;68(18):5117–27. doi: 10.1093/jxb/erx328 29036339 PMC5853260

[92] Wu R, Lucke M, Jang Y-T, Zhu W, Symeonidi E, Wang C, et al. An efficient CRISPR vector toolbox for engineering large deletions in Arabidopsis thaliana. Plant Methods. 2018;14:65. doi: 10.1186/s13007-018-0330-7 30083222 PMC6071326

[93] Xing H-L, Dong L, Wang Z-P, Zhang H-Y, Han C-Y, Liu B, et al. A CRISPR/Cas9 toolkit for multiplex genome editing in plants. BMC Plant Biol. 2014;14:327. doi: 10.1186/s12870-014-0327-y 25432517 PMC4262988

[94] Schindelin J, Arganda-Carreras I, Frise E, Kaynig V, Longair M, Pietzsch T, et al. Fiji: an open-source platform for biological-image analysis. Nat Methods. 2012;9(7):676–82. doi: 10.1038/nmeth.2019 22743772 PMC3855844

