# Supplementary material for: The Arabidopsis thaliana core splicing factor PORCUPINE/SmE1 requires intron-mediated expression
Source: PLoS One. 2025 Mar 26;20(3):e0318163. doi: 10.1371/journal.pone.0318163 (PMC11940714; doi:10.1371/journal.pone.0318163)
Supplement: S1 Method — (DOCX) [file pone.0318163.s001.docx]

**S1 Method.** **Command line instructions used for phylogenetic analysis.**

iqtree -s alignment_file -p partition_file.nex -m MFP -b 100

#nexus

begin sets;

charset part1 = 1-267\\1,2;

charset part2 = 1 - 267\\3;

end;

# where -m MFP is a command that automatically determines best-fit model for the input data

# -b is a bootstrap command

# and nexus parameter commands to search for the best-fitting model of evolution for positions 1 and 2 and position 3 of the codon separately
