# Supplementary figures and images for: The Arabidopsis thaliana core splicing factor PORCUPINE/SmE1 requires intron-mediated expression
Source: PLoS One. 2025 Mar 26;20(3):e0318163. doi: 10.1371/journal.pone.0318163 (PMC11940714; doi:10.1371/journal.pone.0318163)

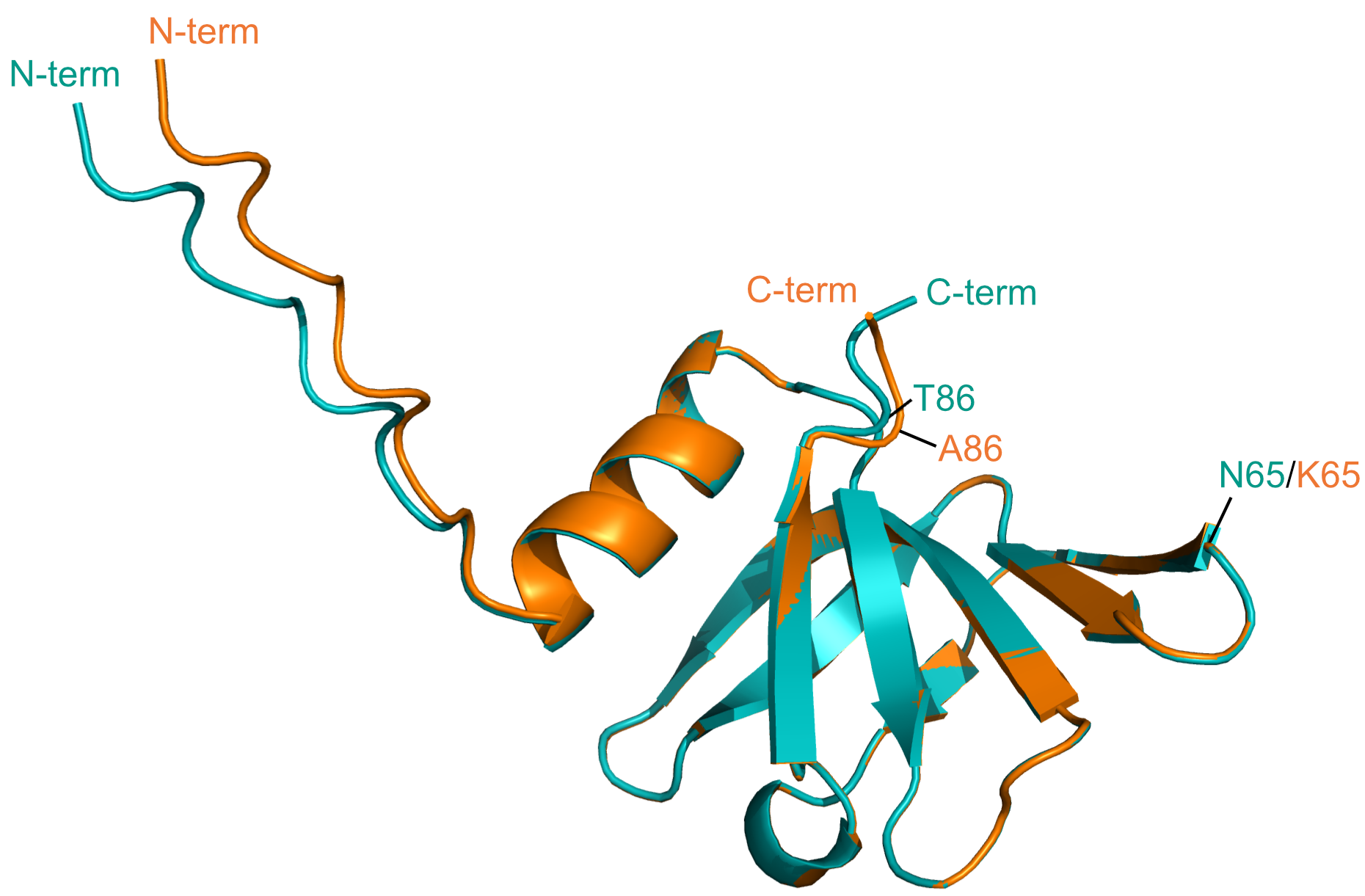

Supplement: S1 Fig — Overlay of PCP (SmE1) (teal) and PCPL (SmE2) (orange) illustrates the high similarity of the predicted structures. N- and C-terminals of the proteins and amino acids in positions 65 and 86 are marked on the protein structures with the corresponding colors. (TIF) [file pone.0318163.s002.tif]

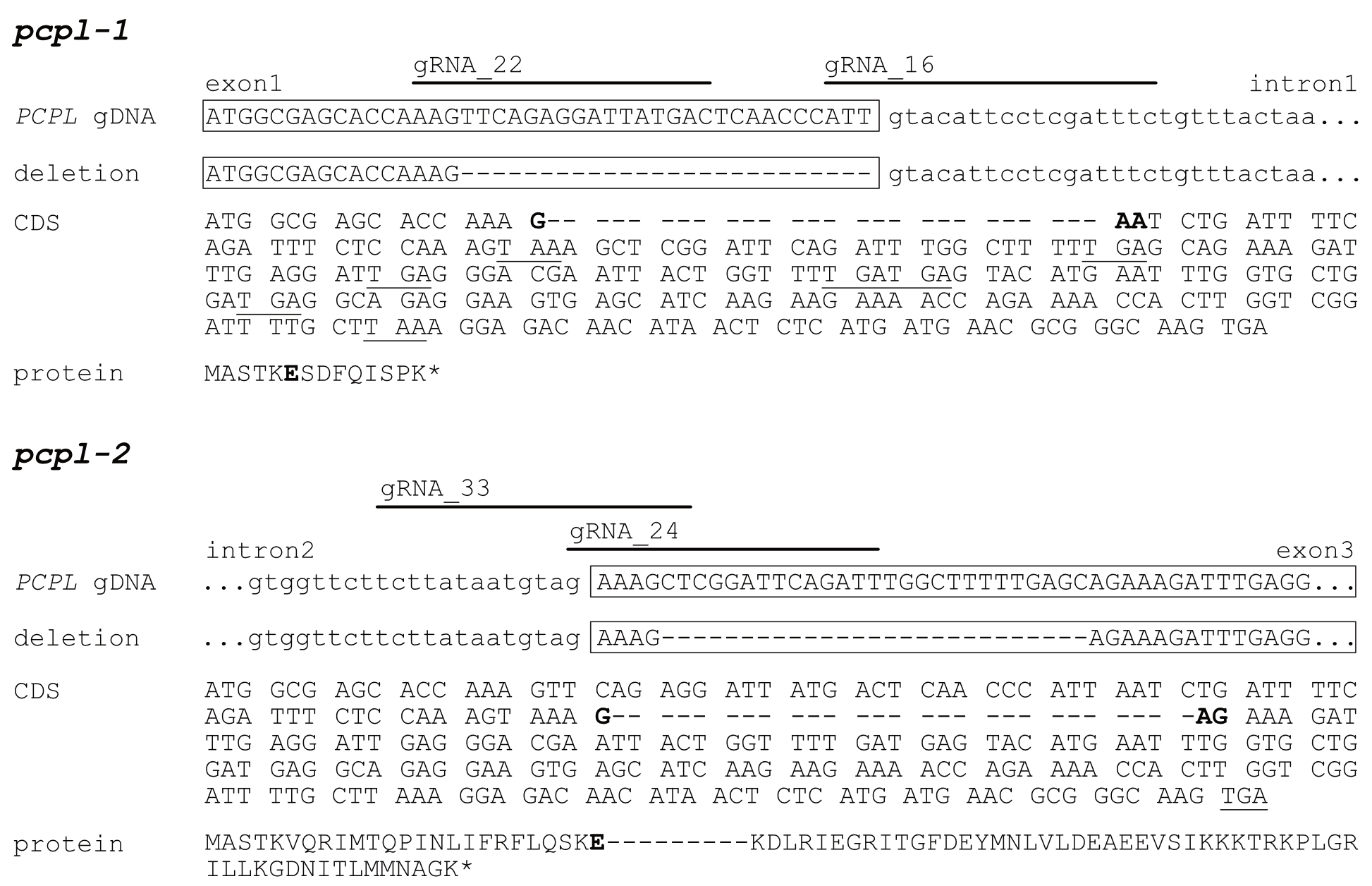

Supplement: S2 Fig — Shown are the region of the genomic PCPL sequence (PCPL gDNA) into which a mutation (deletion) was introduced. Boxes mark exon sequence and dashes indicate the deleted nucleotides. Lines above the gDNA indicate the position of sgRNAs. Underlined letters mark the in-frame stop codons generated in the truncated PCPL coding sequence (CDS). The predicted protein sequence is shown at the bottom with bold letters indicating the first altered amino acid. (TIF) [file pone.0318163.s003.tif]

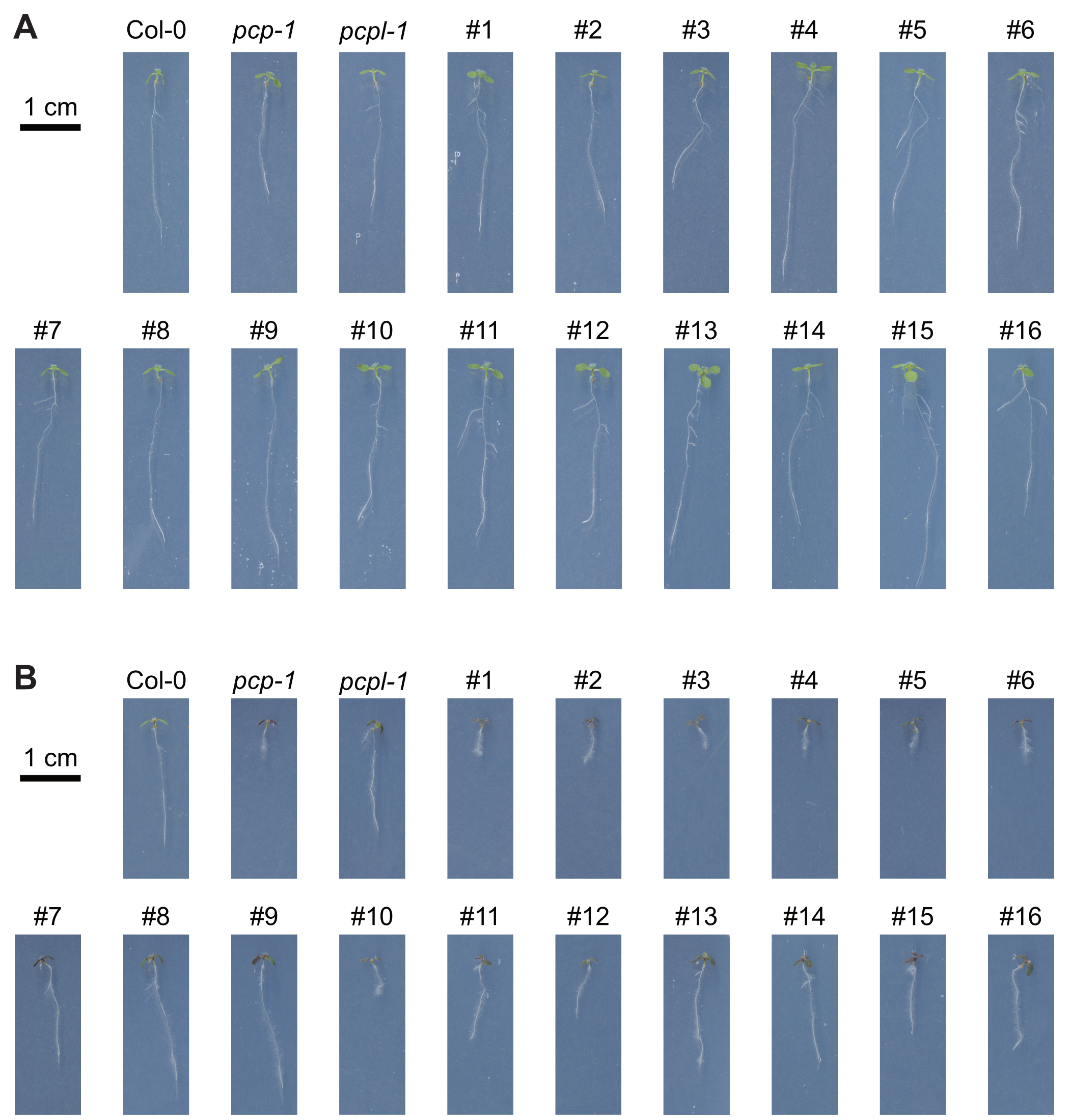

Supplement: S3 Fig — Representative images of the 7-day-old seedlings transformed with various rescue constructs grown at 27°C (A) and 25-day-old seedlings grown at 10°C (B). Three independent lines are shown for each rescue construct generated in this study: #1-3: pPCP::cPCP::tPCP; #4-6: pPCP::cPCPL::tPCP; #7-9: pPCP::gPCP::tPCP; #10-12: pPCP::gPCPL::tPCP; #13: 35S::cPCP::tRbcS [32]; #14-16: 35S::cPCPL::tRbcS. All constructs were expressed in the pcp-1 background. (TIF) [file pone.0318163.s004.tif]

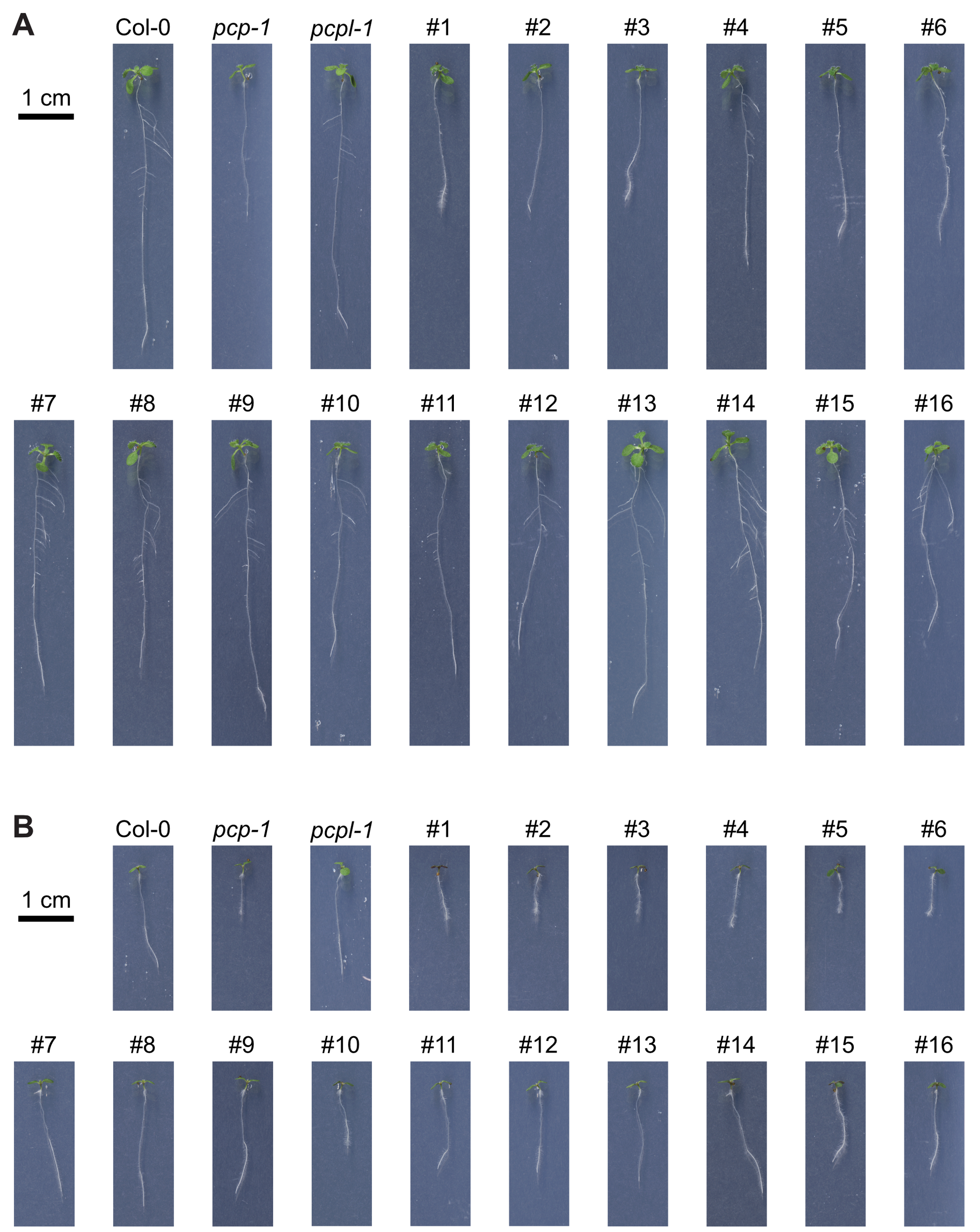

Supplement: S4 Fig — Representative images of the 10-day-old seedlings transformed with various rescue constructs grown at 23°C (A) and 16°C (B). Three independent lines are shown for each rescue construct generated in this study: #1-3: pPCP::cPCP::tPCP; #4-6: pPCP::cPCPL::tPCP; #7-9: pPCP::gPCP::tPCP; #10-12: pPCP::gPCPL::tPCP; #13: 35S::cPCP::tRbcS [32]; #14-16: 35S::cPCPL::tRbcS. All constructs were expressed in the pcp-1 background. (TIF) [file pone.0318163.s005.tif]

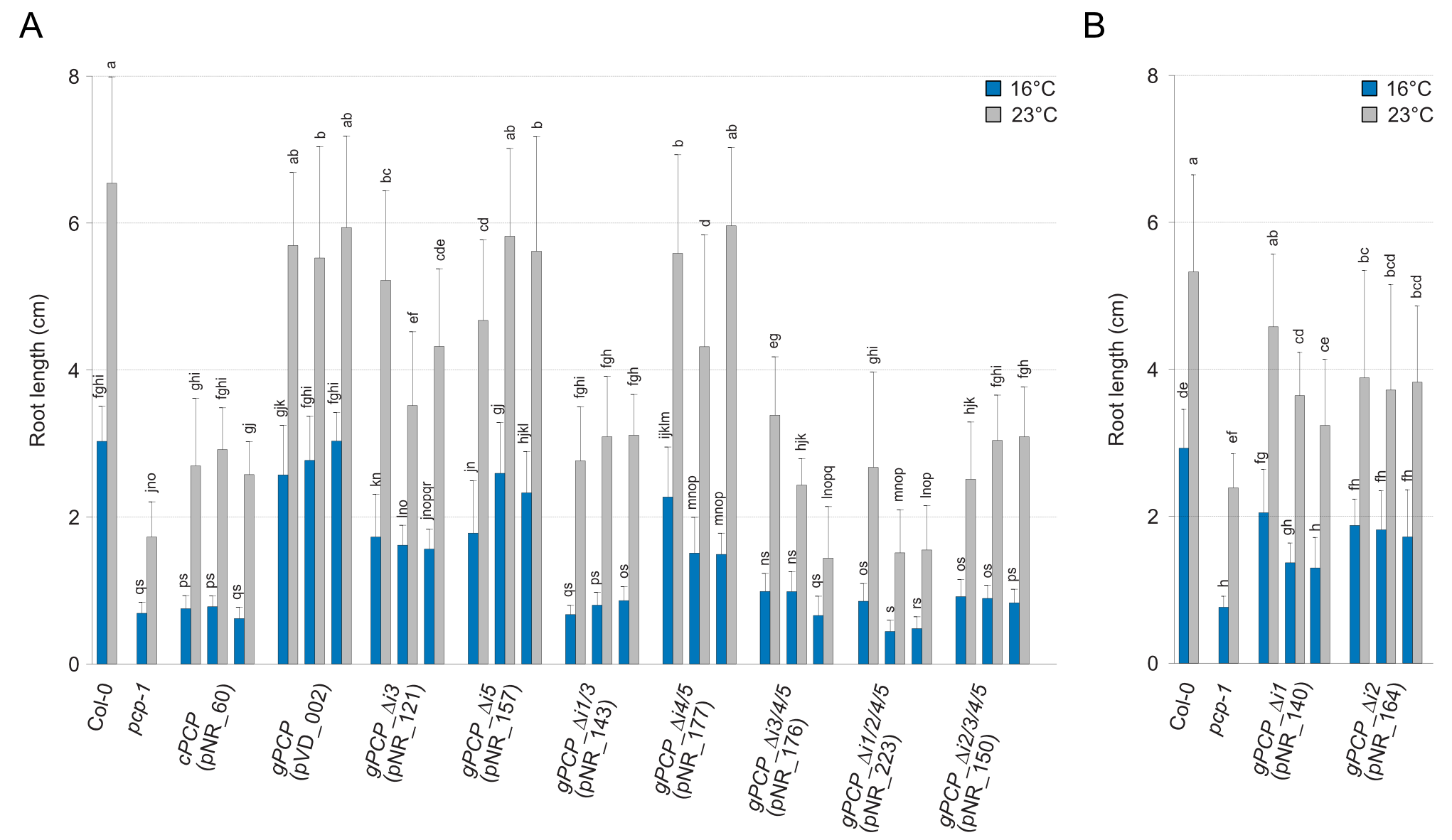

Supplement: S5 Fig — Length of the primary root of three independent lines in the pcp-1 background transformed with various intron deletion constructs. Deletion lines and constructs are labeled as gPCP_ ∆ iX, where gPCP represents a full pPCP::gPCP::tPCP construct, ∆ stands for deletion, i stands for intron, and X indicates the number(s) of the deleted intron(s). Col-0, pcp-1, and a pcp-1 rescue line carrying the full-length pPCP::gPCP::tPCP construct serve as controls. Root length was determined using 10-day-old seedlings grown at 16°C (blue) and 23°C (light grey) for three independent transgenic lines per intron-deletion construct. Bars show the mean of the root length measurement (n = 10 to 37), and error bars indicate the standard deviation (SD). A and B show independent measurements. A two-way ANOVA test with Tukey correction was performed using GraphPad Prism. Letters represent significantly different (P < 0.05) groups. (TIF) [file pone.0318163.s006.tif]

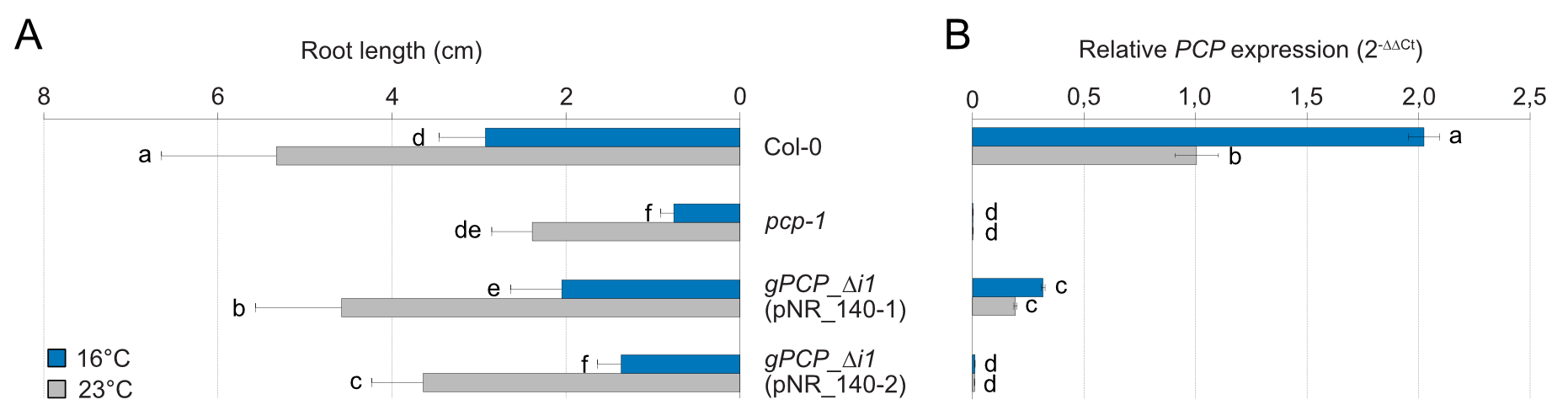

Supplement: S6 Fig — A) Difference in the length of the primary root of independent gPCP_ ∆ i1 lines in the pcp-1 background. Col-0 and pcp-1 serve as controls. Root length was determined using 10-day-old seedlings grown at 16°C (blue) and 23°C (light grey). Bars show the mean of the root length measurement (n = 10 to 28), and error bars indicate the standard deviation (SD). B) Corresponding expression of PCP. Gene expression was determined in seedlings after 5 days of growth at 23°C followed by 24 h of growth at 16°C (blue) or 23°C (grey). Bars show the mean expression calculated from three biological replicates with three technical replicates each. Error bars indicate the standard deviation (SD). A two-way ANOVA test with Tukey correction was performed using GraphPad Prism. Letters represent significantly different (P < 0.05) groups. (TIF) [file pone.0318163.s007.tif]
