# Supplementary material for: The Arabidopsis thaliana core splicing factor PORCUPINE/SmE1 requires intron-mediated expression
Source: PLoS One. 2025 Mar 26;20(3):e0318163. doi: 10.1371/journal.pone.0318163 (PMC11940714; doi:10.1371/journal.pone.0318163)
Supplement: S1 Table — (DOCX) [file pone.0318163.s008.docx]

**S1 Table. *SmE* genes used in phylogenetic analysis.**

| Clade | Species | Gene ID on the phylogenetic tree | Gene Identifier |
| --- | --- | --- | --- |
| Green Algae | *Chlamydomonas reinhardtii* | *Chlamydomonas reinhardtii* | CHLRE_08g385200v5 |
| Bryophytes | *Physcomitrium patens* | *Physcomitrium patens* 1 *Physcomitrium patens* 2 | LOC112292594 LOC112291526 |
| Lycophytes | *Selaginella moellendorffii* | *Selaginella moellendorffii* 1 *Selaginella moellendorffii* 2 | LOC9657844 LOC9634091 |
| Ferns | *Ceratopteris richardii* | *Ceratopteris richardii* | KP509_12G097200 |
| Angiosperms, Amborellaceae | *Amborella trichopoda* | *Amborella trichopoda* 1 *Amborella trichopoda* 2 | LOC18443065 LOC18435458 |
| Monocots, Poaceae | *Brachypodium distachyon* | *Brachypodium distachyon* 1 *Brachypodium distachyon* 2 | LOC100836785 LOC100821943 |
| Monocots, Musaceae | *Musa acuminata* | *Musa acuminata* 1 *Musa acuminata* 2 | LOC103972302 LOC103970691 |
| Monocots, Poaceae | *Oryza sativa* | *Oryza sativa* 1 *Oryza sativa* 2 | LOC4328158 LOC4344666 |
| Monocots, Poaceae | *Panicum hallii* | *Panicum hallii* 1 *Panicum hallii* 2 | LOC112880646 LOC112896754 |
| Eudicots, Rosids, Brassicaceae | *Arabidopsis lyrata* | *Arabidopsis lyrata* 1 *Arabidopsis lyrata* 2 | LOC9303427 LOC9322333 |
| Eudicots, Rosids, Brassicaceae | *Arabidopsis thaliana* | *AtPCP* *AtPCPL* | AT2G18740 AT4G30330 |
| Eudicots, Rosids, Brassicaceae | *Brassica oleracea* | *Brassica oleracea* 1 *Brassica oleracea* 2 | LOC106304253 LOC106332481 |
| Eudicots, Rosids, Brassicaceae | *Brassica rapa* | *Brassica rapa* 1 *Brassica rapa* 2 | LOC103828371 LOC103874062 |
| Eudicots, Rosids, Brassicaceae | *Camelina sativa* | *Camelina sativa* 1  *Camelina sativa* 2  *Camelina sativa* 3  *Camelina sativa* 4  *Camelina sativa* 5  *Camelina sativa* 6 | LOC104730283 LOC104717165 LOC104721822 LOC104747722 LOC104700755 LOC104767242 |
| Eudicots, Rosids, Brassicaceae | *Capsella rubella* | *Capsella rubella* 1 *Capsella rubella* 2 | LOC17880843 LOC17891205 |
| Eudicots, Asterids | *Coffea arabica* | *Coffea arabica* 1 *Coffea arabica* 2 | LOC113738541 LOC113741607 |
| Eudicots, Asterids | *Erythranthe guttata* | *Erythranthe guttata* 1 *Erythranthe guttata* 2 | LOC105955196 LOC105954456 |
| Eudicots, Rosids | *Glycine max* | *Glycine max* 1  *Glycine max* 2  *Glycine max* 3  *Glycine max* 4 | LOC100807929 LOC100306304 LOC100816107 LOC100500256 |
| Eudicots, Rosids | *Gossypium hirsutum* | *Gossypium hirsutum* 1 *Gossypium hirsutum* 2 | LOC107909182 LOC107928261 |
| Eudicots, Rosids | *Medicago truncatula* | *Medicago truncatula* 1 *Medicago truncatula* 2 | LOC11406496 LOC11427208 |
| Eudicots, Rosids | *Populus trichocarpa* | *Populus trichocarpa* 1 *Populus trichocarpa* 2 | LOC18107772 LOC18100540 |
| Eudicots, Rosids | *Quercus robur* | *Quercus robur* 1 *Quercus robur* 2 | LOC126695604 LOC126715753 |
| Eudicots, Asterids | *Solanum lycopersicum* | *Solanum lycopersicum* 1 *Solanum lycopersicum* 2 | LOC101247333 LOC101258239 |
| Eudicots, Asterids | *Solanum tuberosum* | *Solanum tuberosum* 1 *Solanum tuberosum* 2 | LOC102596544 LOC102600833 |
