# Supplementary material for: The Arabidopsis thaliana core splicing factor PORCUPINE/SmE1 requires intron-mediated expression
Source: PLoS One. 2025 Mar 26;20(3):e0318163. doi: 10.1371/journal.pone.0318163 (PMC11940714; doi:10.1371/journal.pone.0318163)
Supplement: S2 Table — (DOCX) [file pone.0318163.s009.docx]

**S2 Table. GreenGate plant binary plasmids used in this study.**

| Plasmid # | Insert | GreenGate vectors | | | | | | |
| --- | --- | --- | --- | --- | --- | --- | --- | --- |
|  |  | Module A | Module B | Module C | Module D | Module E | Module F | Destination vector |
| *pcp-1* phenotype rescue plasmids | | | | | | | | |
| pVD_025 | pPCP::cPCP::tPCP | pRB_01 | pGGB003 | pVD_001 | pGGD002 | pRB_04 | pGGF001 | pGGZ003 |
| pVD_026 | pPCP::cPCPL::tPCP | pRB_01 | pGGB003 | pVD_003 | pGGD002 | pRB_04 | pGGF001 | pGGZ003 |
| pVD_027 | pPCP::gPCP::tPCP | pRB_01 | pGGB003 | pVD_005 | pGGD002 | pRB_04 | pGGF001 | pGGZ003 |
| pVD_028 | pPCP::gPCPL::tPCP | pRB_01 | pGGB003 | pVD_007 | pGGD002 | pRB_04 | pGGF001 | pGGZ003 |
| pVD_095 | p35S::cPCPL::tRbcS | pGGA004 | pGGB003 | pVD_003 | pGGD002 | pGGE001 | pGGF001 | pGGZ003 |
| pVD_098 | pPCP::gPCP_N65K_T86A::tPCP | pRB_01 | pGGB003 | pVD_083 | pGGD002 | pRB_04 | pGGF001 | pGGZ003 |
| pVD_101 | pPCP::gPCP_N65A_T86G::tPCP | pRB_01 | pGGB003 | pVD_086 | pGGD002 | pRB_04 | pGGF001 | pGGZ003 |
| *PCP* intron deletions | | | | | | | | |
| pNR_60 | pPCP::cPCPα_w/o stop::tPCP | pRB_01 | pGGB003 | pRB_02 | pGGD002 | pRB_04 | pNR_072 | pGGZ003 |
| pNR_140 | pPCP::gPCP_Δintron1_IndigStop::tPCP | pRB_01 | pGGB003 | pNR_139 | pGGD002 | pRB_04 | pNR_072 | pGGZ003 |
| pNR_143 | pPCP::gPCPΔintron 1&3_IndigStop::tPCP | pRB_01 | pGGB003 | pNR_141 | pGGD002 | pRB_04 | pNR_072 | pGGZ003 |
| pNR_150 | pPCP::gPCP_Δintron2,3,4&5_IndigStop::tPCP | pRB_01 | pGGB003 | pNR_142 | pGGD002 | pRB_04 | pNR_072 | pGGZ003 |
| pNR_157 | pPCP::gPCP_Δintron5_IndigStop::tPCP | pRB_01 | pGGB003 | pNR_149 | pGGD002 | pRB_04 | pNR_072 | pGGZ003 |
| pNR_164 | pPCP::gPCP_Δintron2_IndigStop::tPCP | pRB_01 | pGGB003 | pNR_147 | pGGD002 | pRB_04 | pNR_072 | pGGZ003 |
| *PCP-LIKE* CRISPR/Cas9 constructs | | | | | | | | |
| Plasmid # | Insert | Amplicon | | | | | | Destination vector |
| pRB_01CF | CF588_sgRNA_PCPL_g16g22 | PCPL_sgRNA_guide_16_22 | | | | | | CF588 |
| pRB_02CF | CF588_sgRNA_PCPL_g24g33 | PCPL_sgRNA_guide_24_33 | | | | | | CF588 |
