# Supplementary material for: The Arabidopsis thaliana core splicing factor PORCUPINE/SmE1 requires intron-mediated expression
Source: PLoS One. 2025 Mar 26;20(3):e0318163. doi: 10.1371/journal.pone.0318163 (PMC11940714; doi:10.1371/journal.pone.0318163)
Supplement: S3 Table — (DOCX) [file pone.0318163.s010.docx]

**S3 Table. Summary of plasmids cloned by overlap PCR used in this study.**

| Plasmid # | Product | Template | Fwd primer | Rev primer |
| --- | --- | --- | --- | --- |
| pNR_121 | pPCP:gPCP_Δintron3_w/ stop:tPCP | pNR_99 | PCR A, O-1558; PCR B, O-3818. | PCR A, O-3817; PCR B, O-3016 ^(1)^ |
| pNR_176 | pPCP:gPCP_ΔIntron3,4&5_w/o stop:tPCP | PCR A, pNR177; PCR B, pNR60 | PCR A, O-1558; PCR B, O-4329. | PCR A, O-4330; PCR B, O-3016 ^(1)^ |
| pNR_177 | pPCP:cPCP_ΔIntron4&5_w/o stop:tPCP | PCR A and C, pNR_60; PCR B, pNR_69 | PCR A, O-1558; PCR B, O-4332; PCR C, 3818. | PCR A, O-4333; PCR B, O-4331; PCR C, 3016 ^(2)^ |
| pNR_223 | pPCP:gPCP_Δintron1,2,4&5_w/o stop:tPCP | PCR A, pNR60; PCR B, pNR-177 | PCR A, O-1558; PCR B, O-4550. | PCR A, O-4557; PCR B, O-3016 ^(1)^ |

(1) Overlap PCR of fragments A and B with O-1558 and O-3016; (2) Overlap PCR of fragments A, B, and C with O-1558 and O-3016
