# Supplementary material for: The Arabidopsis thaliana core splicing factor PORCUPINE/SmE1 requires intron-mediated expression
Source: PLoS One. 2025 Mar 26;20(3):e0318163. doi: 10.1371/journal.pone.0318163 (PMC11940714; doi:10.1371/journal.pone.0318163)
Supplement: S4 Table — (DOCX) [file pone.0318163.s011.docx]

**S4 Table. Oligonucleotides used in this study.**

| Primer # | Name | Sequence (5’3’) |
| --- | --- | --- |
| *Primers used for cloning of genes and gene fragments:* | | |
| O-1558 | pPCP_At2g18740_GG A-mod Fwd | AACAGGTCTCAACCTCCTACGCTAAAAAATTCTAGGTCTTGG |
| O-1559 | pPCP_At2g18740_GG A-mod Rev | AACAGGTCTCTTGTTGGCTAACGTAGTTGGAAATCTCTC |
| O-1560 | PCPa_At2g18740_CDS_Fwd | AACAGGTCTCAGGCTCAACAATGGCGAGCACCAAAGTTCAAAG |
| O-1561 | PCPa_At2g18740_CDS_ Rev | AACAGGTCTCTCTGACTTTCCCGTGTTCATCATCAGAG |
| O-1568 | PCPL_At4g30330_CDS_ Fwd | AACAGGTCTCAGGCTCAACAATGGCGAGCACCAAAGTTCAG |
| O-1569 | PCPL_At4g30330_CDS_Rev | AACAGGTCTCTCTGACTTGCCCGCGTTCATCATGAG |
| O-1592 | PCPL_sgRNA_16_22_E1_ Fwd | AACAGGTCTCTATTGAAATCGAGGAATGTACAATGTTTTAGAGCTAGAAATAGC |
| O-1593 | PCPL_sgRNA_16_22_E1_Rev | AACAGGTCTCTAAACAAGTTCAGAGGATTATGACCAATCTCTTAGTCGACTCTAC |
| O-1594 | PCPL_sgRNA_24_33_E3_ Fwd | AACAGGTCTCTATTGGAAAGCTCGGATTCAGATTGTTTTAGAGCTAGAAATAGC |
| O-1595 | PCPL_sgRNA_24_33_E3_Rev | AACAGGTCTCTAAACAGCTTTCTACATTATAAGACAATCTCTTAGTCGACTCTAC |
| O-2441 | C_cPCPa_At2g18740_D_ Rev | AACAGGTCTCTCTGATCACTTTCCCGTGTTCATCATCAGAG |
| O-2444 | C_cPCP-like_At4g30330_D_ Rev | AACAGGTCTCTCTGATCACTTGCCCGCGTTCATCATGAG |
| O-2500 | C_gPCPa_At2g18740_exon6_D_ Rev | AACAGGTCTCTCTGATCACTTTCCCCTGTTAACATAGAAAGG |
| O-2501 | C_gPCP-like_At4g30330_exon6_D_ Rev | GAACAGGTCTCTCTGATCACTTGCCCCTGTAATAAGATATATAAG |
| O-2626 | pPCP_1.4kb_At2g18740_GG A-mod Fwd | AACAGGTCTCAACCTTTCAATCTTTGTAATTCTAGCATTGAGCACATAGATGTGATTC |
| O-2948 | pPCP1.4kb+gPCPw/o Stop_At2g18740_GG C-mod_ Fwd | AACAGGTCTCAGGCTTTCAATCTTTGTAATTCTAGCATTGAGCACATAGATGTGATTC |
| O-3015 | pAT2S3_GG F mod Fwd | AACAGGTCTCAACTATCAGACCTAAGCTGGCACAACT |
| O-3016 | tMAS_GG F mod Rev | AACAGGTCTCTATACGATAATTTATTTGAAAATTCATAAGAAAAGCAAACG |
| O-3025 | tPCP_At2g18740_GG C-mod Rev | AACAGGTCTCTCTGACAGATTATATTCTGATTGGTCTATTAGTATAATTA |
| O-3742 | gPCP_At2g18740_3' overlap GFP_Rev | GATGCGATCGCACTAGCGGCACCTGACTTTCCCCTGTTAACATAGAAAGG |
| O-3743 | GFP 5'_overlap gPCP_Fwd | TCAGGTGCCGCTAGTGCGATCGCATCAGGG |
| O-3744 | tPCP 3'_Rev | TAGTCAGATTATATTCTGATTGGTCTATTAGTATAATTA |
| O-3745 | mCherry cassette 5'_overlap tPCP_Fwd | ACCAATCAGAATATAATCTGACTATCAGACCTAAGCTGGCACAAC |
| O-3817 | PCP exon3 3'_overlap exon4_Rev | CTAGATTCATGTATTCGTCAAAACCAGTGATTCTTCCTTCAATCCTCAAATCTTTCTGC |
| O-3818 | PCP exon4 5'_overlap exon 3_Fwd | GGTTTTGACGAATACATGAATCTAG |
| O-3819 | gPCP_N65K_Rev_1 | CCATAAGCAGACAGATCTGAGACTTGTTATCAAAGAGTGAGTTCAGGATAACTCACCAAGTGGTTTCCTGGTTTTCTTCTTGATGC |
| O-3820 | gPCP_Rev_2 | CAGAGTTATGTTGTCTCCTTTGAGTAAAATCCTTCCTGAAAATAGGTATTAACACGAGTTATCAAACCATAAGCAGACAGATCTG |
| O-3821 | gPCP_T86A_Rev_3 | GACAAATCAAGCTGCTATACATCATAAAGAGAGAAGACAGAGTAGAGATTGTGTTACTTACGCGTTCATCATCAGAGTTATGTTGTCTCC |
| O-3823 | gPCP_Rev | AACAGGTCTCTCTGATCACTTTCCCCTGTTAACATAGAAAGGATTTTGTTTCAATCTTTTGTTATGTGACAAATCAAGCTGCTATACATC |
| O-3900 | gPCP_N65A_Rev_1 | CCATAAGCAGACAGATCTGAGACTTGTTATCAAAGAGTGAGTTCAGGATAACTCACCAAGTGGTTTCCTGGTAGCCTTCTTGATGC |
| O-3901 | gPCP_T86G_Rev_3 | GACAAATCAAGCTGCTATACATCATAAAGAGAGAAGACAGAGTAGAGATTGTGTTACTTACCCGTTCATCATCAGAGTTATGTTGTCTCC |
| O-3991 | PCP deletion intron1_GG C-mod_Fwd | AACAGGTCTCAACCTAAATCAAGAGAAAATGTGATTCTCGG |
| O-3992 | PCP exon2_ Rev | ACTTTGAAGAAACCTAAAAATCAAGTT |
| O-3993 | PCP exon2_Fwd | AACTTGATTTTTAGGTTTCTTCAAAGT |
| O-4140 | PCP exon3 5'_overlap exon 2_Fwd | AACTTGATTTTTAGGTTTCTTCAAAGTAAAGCTAGGATCCAGATTTGGCTATTTGAG |
| O-4141 | PCP exon4 3'_overlap exon 5_Rev | GTTGTCTCCTTTGAGTAAAATCCTTCCAAGTGGTTTCCTGGTGTTCTTCTTG |
| O-4142 | PCP exon 5_Fwd | GAAGGATTTTACTCAAAGGAGACAACATAAC |
| O-4329 | PCP exon3 5'_overlap intron 2_Fwd | CCTGGTCTTTTGTGTTTGTGATGCAGAAAGCTAGGATCCAGATTTGGCTATTTGAG |
| O-4330 | PCP intron 2 3'_Rev | CTGCATCACAAACACAAAAGACCAGG |
| O-4331 | PCP intron 3_overlap exon 4 5'_Rev | CCAAAACTAGATTCATGTATTCGTCAAAACCCTGCAAAGGACGACGGATAAGCAAGG |
| O-4332 | PCP exon 1 5'_overlap pGG B003 insert_Fwd | AACAGTATTCAGTCGACTGGTACCAACAGGCTCAACAATGGCGAGCACCAAAGTTCAAAG |
| O-4333 | PCP promoter 3'_overlap pGG B003 insert_Rev | AGCCTGTTGGTACCAGTCGACTGAATACTGTTGGCTAACGTAGTTGGAAATCTCTC |
| O-4550 | PCP intron3 5'_Fwd | GTAAGTTGATGTTGAAGCTCCG |
| O-4557 | PCP exon3_overlap intron 3_Rev | CGGAGCTTCAACATCAACTTACAGTGATTCTTCCTTCAATCCTCAAATCTTTCTGC |
| *Sequencing primers:* | | |
| O-0113 | SP6_promoter | AGCTATTTAGGTGACACTATAGAA |
| O-0114 | T7_promoter | GTAATACGACTCACTATAGGGCGA |
| O-0119 | GG-pZ001/pZ003_RB | GTTTACCCGCCAATATATCCT |
| O-0120 | GG-pZ001/pZ003_LB | TTGGCAGGATATATTGTGGTG |
| O-0129 | tRBCS Rev | TCCATTTCCATTTCACAGTTCGA |
| O-0560 | pGGZ003 RB to insert 5’ | TACCCGCCAATATATCCTGTC |
| O-0562 | pGGZ003 LB to insert 3’ | GTGGCAGGATATATTGTGGTG |
| O-1555 | PCP intron 3_Rev | GCCGAAGAGAATGACACAATC |
| O-1564 | PCPa_At2g18740_3'UTR_terminator_Rev | AACAGGTCTCTTAGTCAGATTATATTCTGATTGGTCTATTAGTATAATTA |
| O-1568 | PCPL_At4g30330_CDS_ Fwd | AACAGGTCTCAGGCTCAACAATGGCGAGCACCAAAGTTCAG |
| O-1573 | CF588_Seq_1 | GATCGCTTGGCCTCGC |
| O-1574 | CF588_Seq_2 | CCCTCGTAATTACTTGGGC |
| O-1575 | CF588_Seq_3 Rev | GGTGTGTGCGCAATGAAAC |
| O-1576 | CF588_Seq_4 | TAGCATGCATAAGCGGAGC |
| O-1784 | pGGZ003 to insert 5’ | CGCAACCTTAATTAAACACG |
| O-1801 | PCP exon 1_Fwd | ATGGCGAGCACCAAAGTTCAAAGG |
| O-1817 | pMAS_Rev | GCCCGGTTGCCATGTCCTAC |
| O-2500 | C_gPCPa_At2g18740_exon6_D_ Rev | AACAGGTCTCTCTGATCACTTTCCCCTGTTAACATAGAAAGG |
| O-2504 | PCP exon 4_Fwd | GGATGAGGCTGAAGAAGTGAGCATC |
| O-3427 | pAT2S3_Fwd | TACCACTCATCATAGCTCCG |
