# Supplementary material for: The Arabidopsis thaliana core splicing factor PORCUPINE/SmE1 requires intron-mediated expression
Source: PLoS One. 2025 Mar 26;20(3):e0318163. doi: 10.1371/journal.pone.0318163 (PMC11940714; doi:10.1371/journal.pone.0318163)
Supplement: S5 Table — (DOCX) [file pone.0318163.s012.docx]

**S5 Table. Summary of GreenGate entry modules generated in this study, including information on PCR primers used for amplification.**

| Plasmid # | Product | Template | Fwd primer | Rev primer |
| --- | --- | --- | --- | --- |
| A-modules | | | | |
| pRB_01 | pPCP | Col-0 gDNA | O-1558 | O-1559 |
| C-modules | | | | |
| pRB_02 | cPCP w/o stop codon | Col-0 cDNA | O-1560 | O-1561 |
| pVD_001 | cPCPα w/o stop codon | Col-0 cDNA | O-1560 | O-1561 |
| pVD_002 | cPCPα w/ stop codon | Col-0 cDNA | O-1560 | O-2441 |
| pVD_003 | gPCP w/o stop codon | Col-0 gDNA | O-1560 | O-1562 |
| pVD_004 | gPCP w/ stop codon | Col-0 gDNA | O-1560 | O-2500 |
| pVD_005 | cPCPL w/o stop codon | Col-0 cDNA | O-1568 | O-1569 |
| pVD_006 | cPCPL w/ stop codon | Col-0 cDNA | O-1568 | O-2444 |
| pVD_007 | gPCPL w/o stop codon | Col-0 gDNA | O-1568 | O-1570 |
| pVD_008 | gPCPL w/ stop codon | Col-0 gDNA | O-1568 | O-2501 |
| pVD_083 | gPCP_N65K_T86A | pVD_007 | O-1560 | O-3819, O-3820, O-3821, O-3823 |
| pVD_086 | gPCP_N65A_T86G | pVD_007 | O-1560 | O-3900, O-3820, O-3901, O-3823 |
| pNR_69 | pPCP_1400__gPCP+tPCP | Col-0 gDNA | O-2948 | O-3025 |
| pNR_129 | gPCP_Δintron3_w/o stop | pNR_121 | O-1560 | O-1562 |
| pNR_139 | gPCP_Δintron1_w/ stop | pNR_69 | O-3991 | O-2500 |
| pNR_141 | gPCP_Δintron 1&3_w/ stop | pNR129 | O-3991 | O-2500 |
| pNR_142 | gPCP_ΔIntron2,3,4&5_w/ stop | PCR A, pNR_69; PCR B, pNR_60 | PCR A, O-1560; PCR B, O-3993 | PCR A, O-3992; PCR B, O-2441 ^(2)^ |
| pNR_147 | gPCP_Δintron 2_w/ stop | pNR_69 | PCR A, O-1560; PCR B, O-4140 | PCR A, O-3992; PCR B, O-2500 ^(1)^ |
| pNR_148 | gPCP_Δintron4_w/ stop | pNR_69 | PCR A, O-1560; PCR B, O-4142 | PCR A, O-4141; PCR B, O-2500 ^(1)^ |
| pNR_149 | gPCP_Δintron5_w/ stop | pNR_69 | O-1560 | O-2441 |
| pNR_178 | gPCP_ΔIntron4&5_w/o stop | pNR_177 | O-1560 | O-1561 |
| pNR_222 | gPCP_ΔIntron1,2,4&5_w/o stop | pNR223 | O-1560 | O-1561 |
| pNR_246 | gPCP_Δintron 1,2,3&4_w/ stop | PCR A, pNR_60; PCR B, pNR_69 | PCR A, O-1560; PCR B, O-4142 | PCR A, O-4141; PCR B, O-2500 ^(1)^ |
| E-modules | | | | |
| pRB_04 | tPCP | Col-0 gDNA | O-1563 | O-1564 |
| F-modules | | | | |
| pNR_72 | pAT2S3::mCherry:tMAS | pRW_003 ^(3)^ | O-3015 | O-3016 |

(1) Overlap PCR of fragments A and B with O-1560 and O-2500. (2) Overlap PCR of fragments A and B with O-1560 and O-2441. (3) Wu *et al.*, 2018.
