# Supplementary material for: The Arabidopsis thaliana core splicing factor PORCUPINE/SmE1 requires intron-mediated expression
Source: PLoS One. 2025 Mar 26;20(3):e0318163. doi: 10.1371/journal.pone.0318163 (PMC11940714; doi:10.1371/journal.pone.0318163)
Supplement: S6 Table — (DOCX) [file pone.0318163.s013.docx]

**S6 Table.**  **Primers used for RT-qPCR.**

| **Gene name** | **Gene ID** | **Primer** | **Direction** | **Sequence 5’-3’** |
| --- | --- | --- | --- | --- |
| *PORCUPINE, PCP* | AT2G18740 | O-4917 | Fwd | GGCGAGCACCAAAGTTCAA |
|  |  | O-4918 | Rev | CAAATAGCCAAATCTGGATCCT |
| *PORCUPINE LIKE, PCPL* | AT4G30330 | O-4915 | Fwd | ATGGCGAGCACCAAAGTTCAG |
|  |  | O-4916 | Rev | CAAAAAGCCAAATCTGAATCCG |
| *TUBULIN BETA CHAIN 2, TUB2* | AT5G62690 | O-4807 | Fwd | GAGCCTTACAACGCTACTCTGTCTGTC |
|  |  | O-4808 | Rev | ACACCAGACATAGTAGCAGAAATCAAG |
